# Antineoplastic agent-associated interstitial lung disease in breast, ovarian, and prostate cancers: a pharmacovigilance study using the FDA adverse event reporting system

**DOI:** 10.3389/fimmu.2026.1840323

**Published:** 2026-06-10

**Authors:** Keyuan Du, Chenglong Duan, Jiaqi Zhang, Jianing Zhang, Jinsui Du, Yi Pan, Zhihao Liu, Chenrong Zhang, Yuhan Zhang, Yibin Zhang, Xuan Zhang, Zhongxia Sheng, Bin Wang, Yu Ren, Lizhe Zhu

**Affiliations:** 1 Department of Breast Surgery, The First Affiliated Hospital of Xi’an Jiaotong University, Xi’an, Shaanxi, China; 2 The First Clinical School of Medicine, Xi’an Jiaotong University, Xi’an, Shaanxi, China

**Keywords:** antineoplastic agents, breast cancer, FDA adverse event reporting system (FAERS), interstitial lung disease (ILD), ovarian cancer, pharmacovigilance, prostate cancer

## Abstract

Interstitial lung disease (ILD) is a potentially fatal adverse effect of anticancer therapy, but comparative ILD reporting associations across antineoplastic agents in sex hormone-sensitive solid tumors (breast, ovarian, and prostate cancers) remain unclear. This study aimed to compare ILD reporting associations among antineoplastic agents used in these malignancies and to provide external clinical context from the published literature. We analyzed FAERS reports of ILD associated with 65 prespecified antineoplastic agents used in patients with sex hormone-sensitive solid tumors. Each drug’s ILD reporting association was evaluated using disproportionality analysis (reporting odds ratios), multivariable regression, and time-to-onset analyses. In addition, we conducted a structured PubMed search through March 15, 2026 for eligible human case reports and case series involving these agents. Among 8,146 FAERS ILD reports, trastuzumab deruxtecan (T-DXd) showed a prominent ILD signal and the highest adjusted reporting association. Some sex hormone-pathway agents, including darolutamide and fulvestrant, also showed elevated ILD reporting associations, and concomitant SHA analyses identified additional signals. Older age and lower body weight were independently associated with ILD reporting. In exploratory complete-case TTO analyses, death-recorded ILD reports showed a shorter median reported TTO than non–death-recorded ILD reports; in FDR-adjusted body-weight–stratified analyses, this pattern was observed in the ≥70 kg subgroup but not in the <70 kg subgroup and should be interpreted as hypothesis-generating. The literature review identified 85 eligible publications comprising 98 case-level records and provided supportive clinical context for the FAERS findings. Overall, ILD reporting associations vary across antineoplastic agents used for sex hormone-sensitive solid tumors, even within the same class. Notably, underemphasized signals were observed for certain SHAs. These findings support early ILD monitoring and attention to patient factors such as age and body weight, while underscoring the need for cautious interpretation and confirmation in prospective and real-world studies.

## Introduction

Interstitial lung disease (ILD) comprises a heterogeneous group of fibrosing lung disorders and is among the most serious adverse events associated with anticancer therapy. As these conditions are characterized by interstitial inflammation and fibrosis, ILD directly impairs gas exchange; patients commonly experience exertional dyspnea, dry cough (in approximately 30% of patients), reduced exercise tolerance, and hypoxemia, with substantial decreases in quality of life (1–3). ILD can also progress to respiratory failure (3). Drug-induced ILD is associated with a high risk of death: real-world data indicate an overall mortality of approximately 18.3% among patients with severe ILD related to anticancer agents (23 deaths among 126 severe cases) (4). In oncology, a widely recognized serious adverse effect of anticancer agents—particularly some targeted therapies and ICIs—is drug-associated ILD. Anticancer agents are major contributors, accounting for approximately 23%–51% of drug-related ILD cases (5). These agents may trigger aberrant inflammatory responses through direct alveolar epithelial injury or immune-mediated mechanisms, culminating in pulmonary fibrosis and, in severe cases, respiratory failure; therefore, respiratory symptoms and oxygenation should be closely monitored during treatment (5).

A growing body of evidence from clinical trials, systematic reviews, and real-world studies shows that multiple classes of anticancer agents are associated with ILD. Gartrell et al. conducted a meta-analysis of 4,242 patients receiving mechanistic target of rapamycin (mTOR) inhibitors and reported an incidence of any-grade pneumonitis of approximately 11% (6); in a review of anti-HER2 therapy for breast cancer, Hackshaw et al. reported that the incidence of ILD in patients treated with regimens containing mTOR inhibitors (e.g., everolimus) ranged from 7.3%–21.4% (7). Mechanistically, mTOR inhibitor–associated ILD remains incompletely understood, but proposed mechanisms include alveolar epithelial injury, enhanced apoptosis, immune-mediated dysregulation, and proinflammatory responses, leading to pulmonary inflammation (8). In addition, systematic reviews suggest that programmed cell death protein 1 (PD-1) and programmed death-ligand 1 (PD-L1) inhibitors are associated with an overall incidence of any-grade ILD of approximately 2.9%, with high-grade events of approximately 1%–1.5% (9–11). Mechanistically, ICIs increase peripheral tolerance, resulting in overactivation and pulmonary infiltration of effector T cells and immune-mediated ILD (12–14). In prostate cancer, pulmonary safety concerns have also been observed in contemporary systemic therapy trials; for example, the phase III KEYNOTE-921 trial of pembrolizumab plus docetaxel in metastatic castration-resistant prostate cancer reported treatment-related deaths due to pneumonitis and interstitial lung disease (15). Several small-molecule targeted agents—such as epidermal growth factor receptor (EGFR) tyrosine kinase inhibitors, BRAF inhibitors, cyclin-dependent kinase 4/6 (CDK4/6) inhibitors, and poly (ADP-ribose) polymerase (PARP) inhibitors—are also associated with ILD (5). Conventional endocrine therapy has rarely been associated with ILD: Zhong et al. described a breast cancer patient who developed organizing pneumonia with a “reversed halo sign” after five months of treatment with tamoxifen following surgery and radiotherapy; the condition improved after drug withdrawal and recurred upon rechallenge, suggesting a causal association with tamoxifen (16). The findings of a nationwide Japanese database study also suggested that postoperative radiotherapy combined with or sequential to endocrine therapy is associated with an increased risk of ILD (17). Newer antibody–drug conjugates (ADCs) have also been associated with an increased risk of ILD: published pooled analyses of trastuzumab deruxtecan (T-DXd) have reported ILD/pneumonitis incidences ranging from approximately 11.7% to 15.4%, with fatal events reported in about 2.2% in one pooled analysis (18, 19). Beyond HER2-directed ADCs, mirvetuximab soravtansine has become an important ADC for folate receptor alpha-positive platinum-resistant ovarian cancer (20), and pneumonitis has been identified as an important safety concern in regulatory review (21). Taken together, from conventional chemotherapy and endocrine therapy to small-molecule targeted agents, ICIs, therapeutic antibodies, and ADCs, diverse anticancer agents used across breast, ovarian, and prostate cancer treatment settings have been associated with ILD/pneumonitis.

Although drug-associated ILD has drawn broad attention from clinicians, prior work on anticancer agent–related ILD has largely focused on single drugs or single tumor types. Most published studies are retrospective with limited sample sizes; systematic evaluations across tumor types and therapeutic pathways remain insufficient. Moreover, within the context of sex hormone–sensitive solid tumors, comprehensive comparisons of the risk of ILD across different agents are lacking. This gap in evidence limits clinicians’ overall understanding of the risk landscape and hinders the development of effective monitoring and prevention strategies. For a group of solid tumors that share a specific biological driver—the sex-hormone axis—it remains unclear whether the risk of ILD associated with certain anticancer therapies is shared across breast, ovarian, and prostate cancers. Notably, these malignancies are prototypical hormone-driven solid tumors that share common mechanisms in tumor initiation and progression (22). These three cancers are also major indications for many of the aforementioned anticancer agents, and their treatment strategies overlap extensively; for example, endocrine therapy, cyclin-dependent kinase 4/6 (CDK4/6) inhibitors, mTOR inhibitors, and multiple chemotherapy regimens are widely used across the three. Thus, considering their shared endocrine oncogenesis and overlapping therapeutic armamentarium, analyzing breast, ovarian, and prostate cancers together as a three-cancer framework of sex hormone–sensitive solid tumors has both biological justification and clinical relevance. This overlap also raises a key question: within the context of sex hormone–sensitive solid tumors, how similar or different are the risks of ILD associated with various anticancer agents, and are the risk signals consistent across breast, ovarian, and prostate cancers? Addressing this question is essential for cross-disciplinary, drug class–based risk alerting and patient management.

Notably, although randomized controlled trials (RCTs) have recognized strengths—stronger causal inference from randomization and control, rigorous bias mitigation, and standardized outcome assessment—their restricted population, relatively short follow-up period, and efficacy-focused design inherently limit their ability to evaluate rare, serious adverse events. Consequently, for low-incidence but clinically severe events such as ILD, RCTs often lack sufficient statistical power. Pharmacovigilance data can help fill this gap. The FDA Adverse Event Reporting System (FAERS) is the U.S. Food and Drug Administration (FDA)’s public database that supports postmarketing safety surveillance of drugs and therapeutic biologics. By aggregating large-scale, real-world safety information, the FAERS compensates for the limitations of clinical trials and serves as a key tool for detecting and evaluating postmarketing reporting signals of rare, serious, or previously unrecognized adverse events, thereby providing crucial support for safety research and regulatory decision-making. Using the FAERS, rare but serious adverse events that are difficult to observe in clinical trials can be captured, presenting early warning signals for regulators and clinicians. In this study, we conducted a pharmacovigilance analysis using the FAERS, focusing on breast, ovarian, and prostate cancers as sex hormone–sensitive solid tumors, and systematically compared the strength and characteristics of ILD reporting signals across anticancer agents. Our objective is to address the limitations of RCT evidence and, through an innovatively designed large-scale data analysis, provide a signal-detection and hypothesis-generating framework for cross-drug and cross-pathway evaluation of ILD reporting patterns in this combined three-cancer framework.

## Material and methods

### Data source

**Figure 1:**
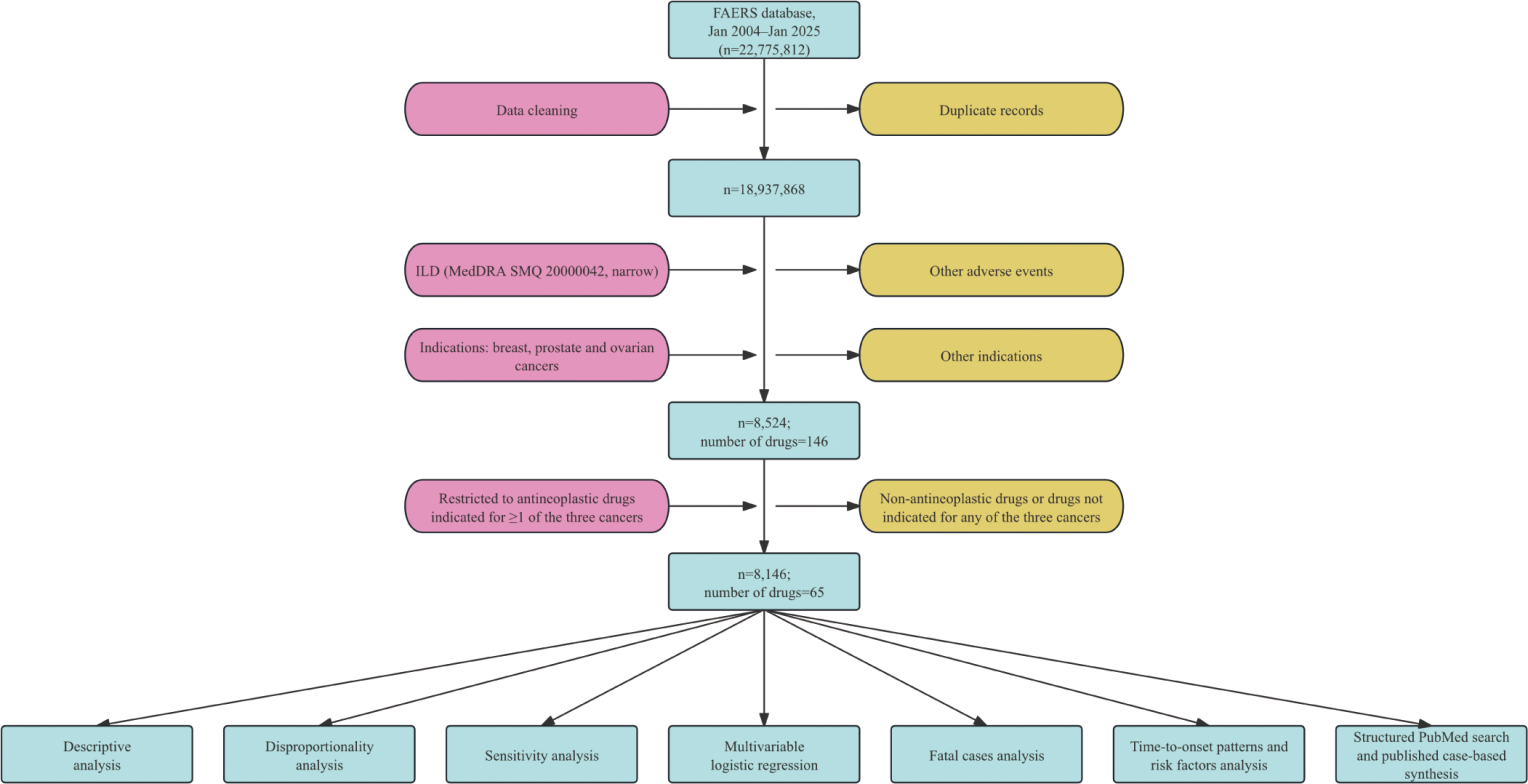
Flow chart of the study. FAERS, FDA Adverse Event Reporting System; ILD, interstitial lung disease; MedDRA, Medical Dictionary for Regulatory Activities; SMQ, Standardized MedDRA Query.

We used data from the FAERS covering 2004 Q1–2025 Q1. Each quarterly release consisted of seven datasets: DEMO (patient demographics and administrative/report information; case level), REAC (adverse events coded by Medical Dictionary for Regulatory Activities [MedDRA] preferred terms [PTs]), DRUG (drug exposure), OUTC (patient outcomes), RPSR (report sources), THER (therapy start/stop dates), and INDI (indications coded by MedDRA). All datasets were imported into PostgreSQL for integrated analyses. Data cleaning and deduplication were performed in accordance with FDA recommendations, and the detailed procedures are provided in the **Supplementary Data Sheet 1**. Adverse events in REAC were coded using MedDRA PTs. ILD was identified with the standardized MedDRA Query (SMQ) “Interstitial lung disease” (code 20000042; narrow scope). Representative PTs in the narrow set included, for example, acute interstitial pneumonitis, bronchiolitis obliterans syndrome, diffuse alveolar damage, and hypersensitivity pneumonitis; the complete list of PTs is provided in **Supplementary Table 1**. We included all antineoplastic agents used for at least one of these three cancers that appeared in the filtered dataset. A case was included in the analysis if the drug of interest was labeled as the primary suspect (PS) for the ILD event. The FAERS is a publicly available, de-identified database; thus, no institutional review board approval was required. An overview of the analytic workflow is shown in **Figure 1**.

### Statistical analysis

All analyses were performed on a case-drug analytic dataset with ILD as a binary outcome. Unless stated otherwise, a two-sided α=0.05 was used. Multiple testing in volcano plot analyses was controlled with the Benjamini–Hochberg false discovery rate (FDR).

We used a case–noncase approach to calculate RORs for each drug–event pair (**Supplementary Table 2**). A positive disproportionality signal was declared only when both criteria were met: (i) ≥ 4 reports for the pair (23) and (ii) the lower bound of the 95% confidence intervals (CIs) for the ROR > 1. The volcano plot displays ln(ROR) against −log10(q), where q denotes the FDR-adjusted *p* value. We then fitted two multivariable logistic regression models with ILD (yes/no) as the outcome, modeling drug exposure as a 65-level indicator and using capecitabine as the reference drug; the criteria for selecting the reference drug are detailed in the **Supplementary Data Sheet 1**. In Model A (all drugs), covariates included age (years), weight (kg), comedication (yes/no; any concomitant drug recorded in addition to the index exposure), and reporter type (categorical). In Model B (comedication effect of sex hormone–pathway agents [SHAs]), after excluding reports in which an SHA was the primary suspect, we additionally included binary indicators for concomitant SHA use (present/absent), with age and weight entered as continuous covariates and reporter type as a categorical covariate. Because Model B was fitted to a pooled breast, ovarian, and prostate cohort, coefficients represent average associations across the combined population; cancer-specific effects were not targeted and no cancer-stratified analyses were performed. Our primary inferential focus was the independent association of SHAs when used as concomitants. Because spontaneous reporting patterns may vary across calendar years, particularly for drugs approved or monitored during different periods, we further conducted a sensitivity version of Model A with reporting year included as an additional categorical covariate. This analysis was used to examine whether the main drug-level reporting associations were broadly robust to adjustment for reporting year; detailed procedures are provided in the **Supplementary Data Sheet 1**.

Among ILD reports, we additionally performed exploratory analyses of reported onset timing and death-recorded outcomes. Detailed definitions and statistical procedures, including complete-case TTO inclusion/exclusion assessment, and comparison of TTO-included versus TTO-excluded reports, ECDF/Wilcoxon comparisons, stratified analyses, Cox models, Weibull modeling, are provided in the **Supplementary Data Sheet 1**.

Data wrangling, statistical analyses, and graphics were performed in R (version 4.5.0).

### Literature search and study selection

To contextualize the FAERS findings with published clinical evidence, we conducted a structured PubMed search from database inception to March 15, 2026 for eligible human case reports and case series of antineoplastic agent-associated interstitial lung disease (ILD). No publication-year restriction was applied. Two complementary search strategies were used. The first combined ILD-related terms with broad antineoplastic treatment concepts, including chemotherapy, immunotherapy, endocrine therapy, targeted therapy, immune checkpoint inhibitors (ICIs), and antibody-drug conjugates (ADCs). The second combined ILD-related terms with a prespecified list of 65 antineoplastic agents included in the main FAERS analyses. The full search strings are provided in the **Supplementary Data Sheet 2**. Both searches were restricted to human case reports and case series. Search results were merged, and duplicates were removed using PubMed unique identifiers (PMIDs) only. Full texts were then screened for eligibility by two reviewers. Studies were included if they (1) were human case reports or case series, (2) involved patients with breast, ovarian, or prostate cancer, (3) reported exposure to at least one of the 65 prespecified antineoplastic agents, and (4) described ILD, or ILD-consistent pneumonitis/interstitial pneumonia, consistent with the FAERS ILD case definition. Studies were excluded if they were non-human studies, were not case reports or case series, involved cancers other than the three target malignancies without clear identification of eligible cases, did not include any of the prespecified agents or did not allow confirmation of the culprit agent, lacked sufficient clinical information to ascertain cancer type, drug exposure, or ILD, were unavailable in full text, or represented duplicate publication of the same case, in which case the most informative report was retained.

## Results

### Descriptive analysis

A total of 8,146 FAERS reports of interstitial lung disease (ILD) associated with therapy for sex hormone-sensitive solid tumors were included, comprising 6,176 breast cancer, 728 ovarian cancer, and 1,242 prostate cancer reports. Sex distribution aligned with tumor characteristics: most breast and ovarian cancer reports were from females, whereas most prostate cancer reports were from males; the non–100% female proportion in ovarian cancer reflected missing sex information rather than male patients. Overall, the median age was 65 years (interquartile range [IQR] 55–73), with prostate cancer reports skewing older (median 75 years [IQR 70–80]). The median body weight was 64 kg (IQR 53–76). The leading reporting countries were Japan (30.6%) and the United States (22.7%). All outcomes met FAERS seriousness criteria; “other serious” (35.0%) and hospitalization (33.6%) were most frequently reported, and death was recorded in 20.8% overall, with a higher proportion in prostate cancer (36.2%) (**Table 1**).

**Table 1:** Baseline characteristics of ILD following antineoplastic therapy for sex hormone-sensitive solid tumors from the FAERS: overall and by cancer type (breast, ovarian, prostate).

Characteristic	Overall,n(%)	Breast cancer,n(%)	Ovarian cancer,n(%)	Prostate cancer,n(%)
Number of patients	8146	6176	728	1242
Gender				
Data absence	633 (7.77)	469 (7.59)	117 (16.07)	47 (3.78)
Female	6246 (76.68)	5633 (91.21)	611 (83.93)	2 (0.16)
Male	1267 (15.55)	74 (1.20)	0 (0.00)	1193 (96.05)
Age (years)				
Data absence	2429 (29.82)	1897 (30.72)	253 (34.75)	279 (22.46)
≤40	415 (5.09)	358 (5.80)	27 (3.71)	30 (2.42)
40-65	2485 (30.51)	2173 (35.18)	205 (28.16)	107 (8.62)
>65	2817 (34.58)	1748 (28.30)	243 (33.38)	826 (66.51)
Median (IQR)	65(55-73)	62(52-71)	66(57-73)	75(70-80)
Weight (kg)				
Data absence	5230 (64.20)	3935 (63.71)	488 (67.03)	807 (64.98)
<40	72 (0.88)	52 (0.84)	18 (2.47)	2 (0.16)
40–50	407 (5.00)	307 (4.97)	57 (7.83)	43 (3.46)
50–60	699 (8.58)	532 (8.61)	69 (9.48)	98 (7.89)
60–70	658 (8.08)	517 (8.37)	44 (6.04)	97 (7.81)
70–80	514 (6.31)	410 (6.64)	29 (3.98)	75 (6.04)
80–90	289 (3.55)	225 (3.64)	6 (0.82)	58 (4.67)
≥90	277 (3.40)	198 (3.21)	17 (2.34)	62 (4.99)
Median (IQR)	64(53-76)	64(53-76)	56(48-67.25)	67.5(56.75-80)
Reported countries (Top 7 by frequency)				
Data absence	294 (3.61)	211 (3.42)	27 (3.71)	56 (4.51)
Japan	2493 (30.60)	1465 (23.72)	361 (49.59)	667 (53.70)
United States	1845 (22.65)	1546 (25.03)	149 (20.47)	150 (12.08)
France	556 (6.83)	452 (7.32)	38 (5.22)	66 (5.31)
Germany	534 (6.56)	466 (7.55)	13 (1.79)	55 (4.43)
United Kingdom	301 (3.70)	244 (3.95)	16 (2.20)	41 (3.30)
Canada	274 (3.36)	235 (3.81)	11 (1.51)	28 (2.25)
Italy	215 (2.64)	180 (2.91)	29 (3.98)	6 (0.48)
Outcomes				
Data absence	264 (3.24)	250 (4.05)	13 (1.79)	1 (0.08)
Death	1696 (20.82)	1161 (18.80)	85 (11.68)	450 (36.23)
Life-threatening	526 (6.46)	401 (6.49)	42 (5.77)	83 (6.68)
Hospitalization	2736 (33.59)	2092 (33.87)	236 (32.42)	408 (32.85)
Disability	61 (0.75)	45 (0.73)	9 (1.24)	7 (0.56)
Required intervention	15 (0.18)	9 (0.15)	1 (0.14)	5 (0.40)
Other serious	2848 (34.96)	2218 (35.91)	342 (46.98)	288 (23.19)

Notes: Data are summarized as n (%) or median (IQR). Stratifications included sex; geographic region (U.S., Japan, other); and serious outcomes (death, life-threatening, hospitalization, disability, other). Abbreviations: FAERS, FDA Adverse Event Reporting System; ILD, interstitial lung disease; IQR, interquartile range.

### Disproportionality analysis

**Figure 2:**
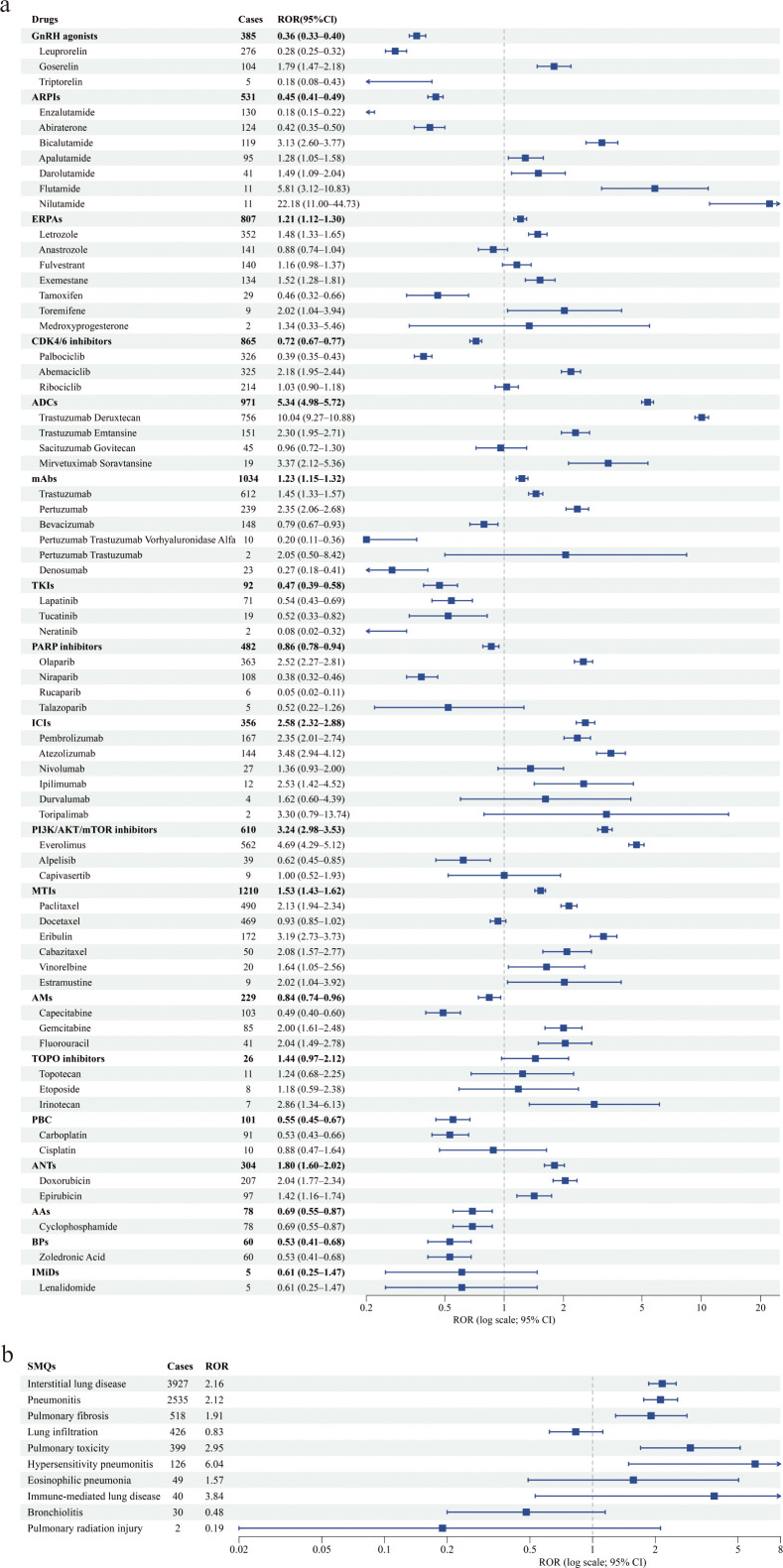
Disproportionality analysis of antineoplastic drugs associated with ILD in patients with sex hormone–sensitive solid tumors. Notes. **(A)** Forest plots of RORs with 95% CIs by pharmacologic class, with specific drugs in each class displayed. **(B)** Forest plot of RORs for ILD-related MedDRA PTs. A positive reporting disproportionality signal was defined as a lower 95% CI bound > 1 with ≥4 coreports; RORs with 95% CIs are displayed on a log scale. Abbreviations: ROR, reporting odds ratio; CI, confidence interval; PT, preferred term; SMQ, standardized MedDRA query; ARPIs, androgen receptor pathway inhibitors; GnRH agonists, gonadotropin-releasing hormone agonists; ERPAs, estrogen/progesterone receptor pathway agents; CDK4/6 inhibitors, cyclin-dependent kinase 4/6 inhibitors; ADCs, antibody–drug conjugates; mAbs, monoclonal antibodies; TKIs, tyrosine kinase inhibitors; PARP inhibitors, poly(ADP-ribose) polymerase inhibitors; ICIs, immune checkpoint inhibitors; PI3K/AKT/mTOR inhibitors, PI3K/AKT/mTOR pathway inhibitors; MTIs, microtubule inhibitors; AMs, antimetabolites; TOPO inhibitors, topoisomerase inhibitors; PBC, platinum-based chemotherapy; ANTs, anthracycline antibiotics; AAs, alkylating agents; BPs, bisphosphonates; IMiDs, immunomodulatory drugs; ILD, interstitial lung disease.

We mapped reporting disproportionality for interstitial lung disease (ILD) across anticancer classes and individual agents used for sex hormone–sensitive solid tumors using the reporting odds ratio (ROR), applying the prespecified signal criterion (lower 95% CI bound > 1 with ≥4 coreports) (**Figure 2A**). At the class level, positive signals were observed for antibody–drug conjugates, immune checkpoint inhibitors, PI3K/AKT/mTOR pathway inhibitors, microtubule inhibitors, anthracyclines, and monoclonal antibodies, whereas tyrosine kinase inhibitors and several cytotoxic classes were not detected. Class-level estimates masked heterogeneous drug-level reporting patterns within pharmacologic classes; for example, androgen receptor pathway inhibitors were not detected overall, but several agents—particularly first-generation nonsteroidal antiandrogens—met the signal criterion, and abemaciclib and olaparib were positive despite their respective classes being not detected overall. We further assessed ILD-related preferred terms, and among the ten most frequently reported terms, five showed positive reporting disproportionality signals—”interstitial lung disease,” “pneumonitis,” “pulmonary fibrosis,” “pulmonary toxicity,” and “hypersensitivity pneumonitis” (**Figure 2B**).

**Figure 3:**
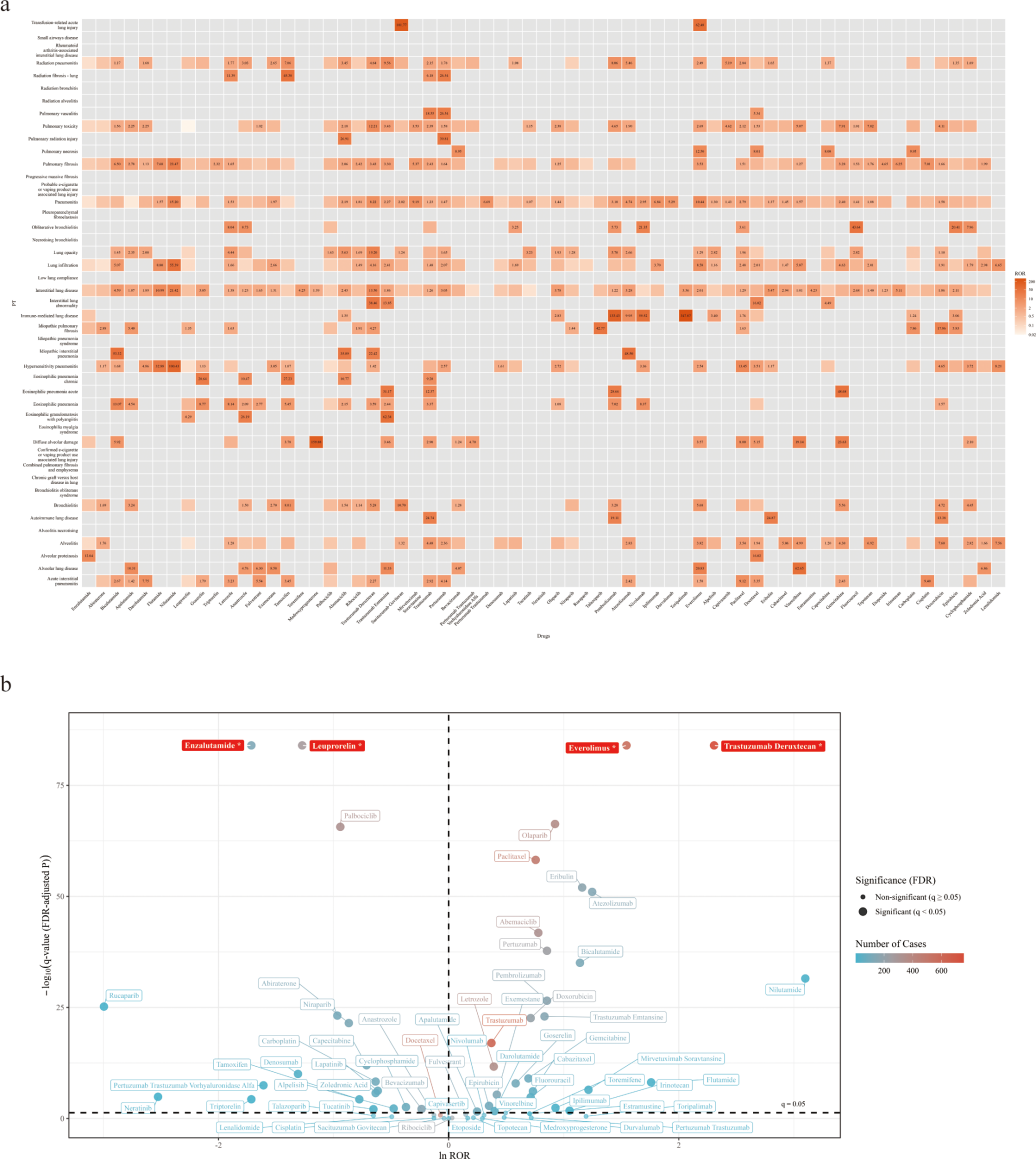
**(A)** Drug–PT heatmap of ILD disproportionality (ROR) in patients with sex hormone-sensitive solid tumors. **(B)** Volcano plot of drug-level ILD reporting signals in patients receiving antineoplastic therapy for sex hormone-sensitive solid tumors. Notes: **(A)** We displayed the drug-by-PT pairs as a heatmap with color indicating the ROR. Numeric labels were shown only when the ROR > 1 to aid visual screening and did not indicate a positive reporting signal. A positive reporting signal was defined as a lower 95% CI bound > 1 with ≥4 coreports. The axes represent drugs (x) and PTs (y). **(B)** Each point represents a drug; the x-axis shows the natural log of the reporting odds ratio (ln ROR), and the y-axis shows the −log10 of the FDR-adjusted *P* (q-value). The point color reflects the number of ILD reports (blue = fewer; red = more), and the point size denotes significance (q<0.05 vs. q≥0.05). The vertical dashed line reflects ln ROR = 0 (no disproportionality), and the horizontal dashed line reflects q=0.05. Drug names are labeled; extremely significant points are highlighted. Analyses included sex hormone pathway agents (SHAs), newer anticancer agents, and traditional chemotherapy/other drugs. Abbreviations: PT, preferred term; ILD, interstitial lung disease; ROR, reporting odds ratio; CI, confidence interval; FDR, false discovery rate; SHAs, sex hormone-pathway agents.

In addition, we visualized the disproportionality pattern using a drug-by-event (PT) heatmap with the ROR as the color scale; numeric values were annotated within a cell only when the ROR was > 1. These annotations were intended for visual screening and did not imply statistical positivity. The heatmap indicated that the most frequent PTs were ILD, pneumonitis, pulmonary fibrosis, lung infiltration, and pulmonary toxicity, with annotations appearing for 33, 31, 26, 24, and 23 drugs, respectively. The drugs covering the greatest number of PTs were everolimus and trastuzumab (15 each), followed by paclitaxel and T-DXd (14 each) and pertuzumab (13) (**Figure 3A**).

Finally, we conducted a volcano plot analysis to evaluate the disproportionality signals between antineoplastic agents used to treat sex hormone-sensitive solid tumors and ILD, grouped by drug class (SHAs, newer anticancer agents, and traditional chemotherapy/other drugs). The association between the ILD reporting signals and the SHAs, including bicalutamide, darolutamide, goserelin, and letrozole, was positive and statistically significant. In contrast, the ILD signals associated with newer anticancer agents (such as targeted therapies, immune therapies, and ADCs) showed a more mixed distribution, although significant positive associations were observed for trastuzumab deruxtecan, abemaciclib, everolimus, and pembrolizumab. Similarly, traditional chemotherapies and other commonly used antineoplastic agents exhibit a mixed pattern of ILD reporting, with significant positive associations for paclitaxel, gemcitabine, doxorubicin, and irinotecan. (**Figure 3B**).

### Multivariable logistic regression

**Figure 4:**
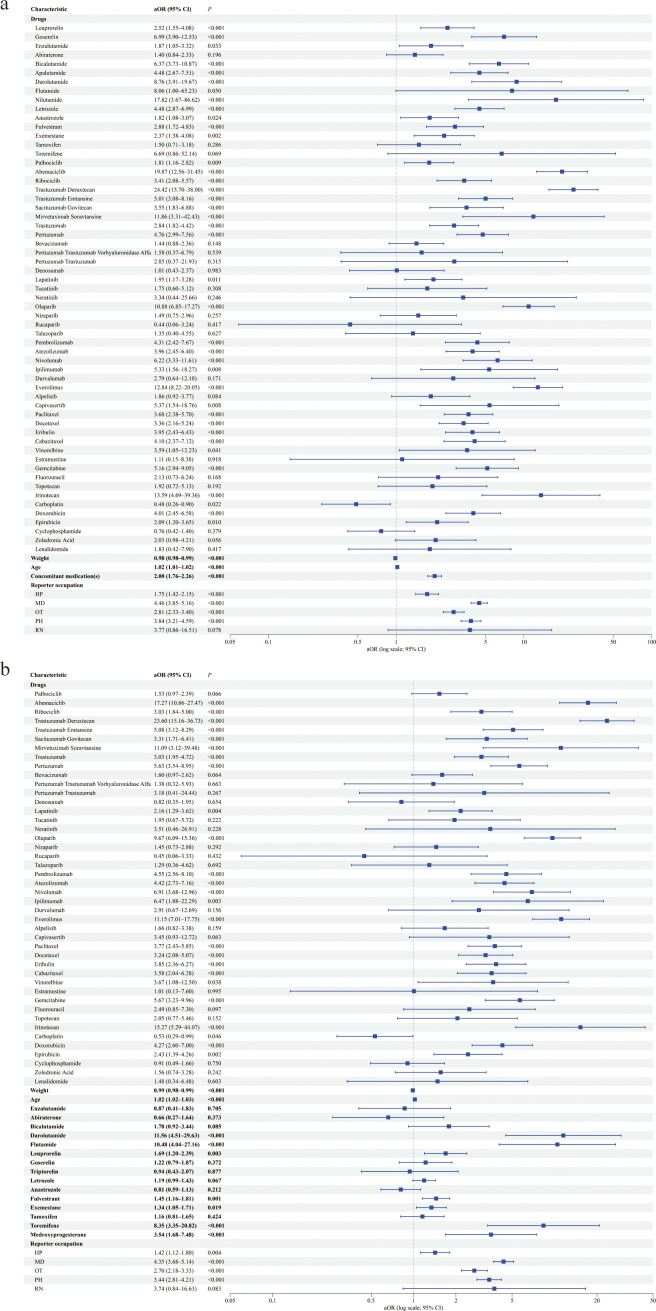
**(A)** Model A: Drug-level aORs for ILD in patients with sex hormone–sensitive solid tumors. **(B)** Model B: Concomitant SHA use—drug-level aORs for ILD in sex hormone–sensitive solid tumors. Notes: **(A)** We performed multivariable logistic regression to assess drug–ILD associations with capecitabine as the reference, adjusting for age, weight, any concomitant medication, and reporter type. The forest plot shows aORs with 95% CIs on a log scale. **(B)** After excluding reports in which an SHA was the primary suspect, we included a binary covariate for concomitant SHA (present vs. absent) and adjusted for age, weight, and reporter type, with capecitabine as the drug reference. The forest plot displays aORs with 95% CIs on a log scale. Abbreviations: aOR, adjusted odds ratio; CI, confidence interval; HP, health professional; MD, physician; OT, other health professional; PH, pharmacist; RN, nurse; CN, consumer; ILD, interstitial lung disease.

Building on the disproportionality analysis, we fitted a multivariable logistic regression model (Model A) using capecitabine as the reference and adjusting for age, weight, comedication, and reporter type. Older age and lower body weight were independently associated with ILD (aOR 1.018 per 1-year increase and 0.985 per 1-kg increase; both *P* < 0.001). Among 64 drugs entered, 37 showed significantly higher adjusted odds of ILD. The largest adjusted reporting association was observed for trastuzumab deruxtecan (T-DXd; aOR 24.42; *P* < 0.001), whereas carboplatin was the only drug associated with lower odds of ILD reporting (aOR 0.48; *P=*0.022). Full drug-specific estimates are presented in **Figure 4A**.

Given that drug-level ILD reporting associations in FAERS may be influenced by calendar-time variation and reporting practices, we performed a reporting-year-adjusted sensitivity analysis based on Model A. The reporting-year-adjusted results were generally consistent with the primary Model A. Of the 37 non-reference drugs that met the positivity criterion in the primary Model A, 34 remained positive after reporting-year adjustment. Representative retained associations included trastuzumab deruxtecan (primary Model A: aOR 24.42; 95% CI 15.70–38.00; reporting-year-adjusted Model A: aOR 27.28; 95% CI 17.41–42.75), abemaciclib (19.87; 95% CI 12.56–31.45 vs. 21.88; 95% CI 13.78–34.74), darolutamide (8.76; 95% CI 3.91–19.67 vs. 8.48; 95% CI 3.76–19.16), and fulvestrant (2.88; 95% CI 1.72–4.83 vs. 2.91; 95% CI 1.74–4.88). In contrast, anastrozole, lapatinib, and vinorelbine no longer met the positivity criterion after reporting-year adjustment, whereas alpelisib became positive. These findings suggest that reporting-year effects may have influenced the magnitude or precision of some drug-specific estimates, but did not fully account for the principal drug-level reporting associations observed in the primary Model A. Full results are provided in **Supplementary Table 3**.

On the basis of Model A, we constructed Model B to assess the independent effect of SHAs on ILD when used concomitantly with other antineoplastic agents. We first excluded reports in which an SHA was the primary suspect, then added a binary covariate for concomitant SHA use and entered age and weight as continuous covariates with reporter type as a categorical covariate. The results revealed that among the GnRH agonists (n = 3), leuprorelin was significantly associated with ILD (aOR 1.69; *P =* 0.003). Among the ARPIs (androgen receptor pathway inhibitors) (n = 7), darolutamide (aOR 11.56; *P <* 0.001) and flutamide (aOR 10.48; *P <* 0.001) were significantly associated with ILD. Among the ERPAs (estrogen/progesterone receptor pathway agents) (n = 7), fulvestrant (aOR 1.45; *P =* 0.001), exemestane (aOR 1.34; *P =* 0.019), toremifene (aOR 8.35; *P <* 0.001), and medroxyprogesterone (aOR 3.54; *P <* 0.001) were significantly associated with ILD. No significant associations were observed for the remaining drugs. (**Figure 4B**).

### Analysis of death-recorded reports

Of the 8,146 ILD reports, 3,464 had valid therapy start and ILD event onset dates with a nonnegative calculated TTO and were included in the complete-case TTO analyses. The remaining 4,682 reports were excluded because of missing therapy start dates, missing event onset dates, missing both dates, or negative TTO values. (**Supplementary Table 4**) Characteristics of TTO-included and TTO-excluded reports are summarized in **Supplementary Table 5**. Several demographic, reporting, tumor-related, outcome-related, and drug-class variables showed imbalance between included and excluded reports based on SMDs, suggesting that date availability was unlikely to be completely random. Therefore, all TTO findings were interpreted as exploratory complete-case reporting-pattern analyses.

**Figure 5:**
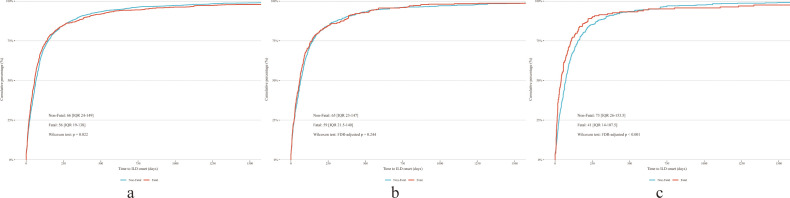
ECDF curves for the time to onset of ILD following antineoplastic therapy for sex hormone-sensitive solid tumors: death-recorded reports vs non–death-recorded reports. Notes: Panels display ECDF curves comparing the distributions of ILD reports with and without recorded death outcomes in **(A)** the overall population, **(B)** patients weighing <70 kg, and **(C)** patients weighing ≥70 kg. Group differences in reported TTO were evaluated using two-sided Wilcoxon rank-sum tests, and the panel-level *P* values shown for body-weight subgroup comparisons are FDR-adjusted *P* values calculated using the Benjamini–Hochberg method. The 70-kg cutoff was used as a clinically interpretable adult reference body-weight cutoff; however, the body-weight subgroup analysis was exploratory, and the ≥70 kg finding should be interpreted as hypothesis-generating rather than confirmatory. Abbreviations: ECDF, empirical cumulative distribution function; FDR, false discovery rate; ILD, interstitial lung disease; IQR, interquartile range; TTO, time to onset.

In the complete-case exploratory TTO analysis, ILD reports with death recorded in the FAERS OUTC field had a shorter median reported TTO than reports without death recorded (56 vs. 66 days; overall *P* = 0.022). In body-weight subgroup analyses, the difference remained statistically significant after FDR correction in the ≥70 kg subgroup (median 41 vs. 73 days; FDR-adjusted *P* < 0.001), whereas no significant difference was observed in the <70 kg subgroup (median 59 vs. 65 days; FDR-adjusted *P* = 0.244) (**Figure 5**). Because the 70-kg cutoff was used as a clinically interpretable adult reference body-weight threshold rather than an ILD-specific clinical risk threshold, this subgroup finding was considered exploratory and hypothesis-generating. In age-stratified analyses, death-recorded reports showed numerically shorter median reported TTO across all age categories, but none of the age-stratified comparisons remained statistically significant after FDR correction (≤40 years: median 42 vs. 66 days, FDR-adjusted *P* = 0.073; >40–≤65 years: median 44 vs. 64 days, FDR-adjusted *P* = 0.061; >65 years: median 56 vs. 70 days, FDR-adjusted *P* = 0.061) (**Supplementary Figure 4**).

### Published case-based clinical context

To provide external clinical context for the FAERS findings, we conducted a structured PubMed search for published human case reports and case series of interstitial lung disease (ILD) involving the prespecified antineoplastic agents. The search identified 3,370 records; 85 publications met the eligibility criteria after screening and full-text review, yielding 98 case-level records for descriptive synthesis. The full list of the 85 eligible source publications is provided in **Supplementary Data Sheet 3**.

Most published cases involved breast cancer (63/98), followed by prostate cancer (20/98) and ovarian cancer (14/98); one additional case involved both breast and ovarian cancer. Patients were predominantly female (77/98), with a median age of 62 years (interquartile range [IQR], 51–72 years). Among 74 cases with extractable time-to-onset data, the median time to ILD onset was 60 days (IQR, 21–128 days). Fatal outcomes were reported in 19 cases.

Among records in which the primary suspect (PS) entry consisted of one or more of the 65 prespecified agents (n = 93), the most frequent PS entries were docetaxel (n = 15), paclitaxel (n = 10), trastuzumab (n = 9), cyclophosphamide (n = 6), and gemcitabine, abemaciclib, and apalutamide (n = 4 each). Among fatal cases, docetaxel was the most frequent PS entry (8/19), followed by cyclophosphamide (2/19); the remaining cases involved trastuzumab, paclitaxel, gemcitabine, doxorubicin, palbociclib, medroxyprogesterone, anastrozole, and combined antiandrogen/GnRH-agonist regimens.

Because sex hormone-pathway agents (SHAs) were the focus of this study, we further summarized cases in which the PS regimen involved an SHA. Overall, 15 of 98 published cases involved an SHA as the PS regimen, including 13 prostate cancer cases and 2 breast cancer cases. Of these, 11 involved a single SHA as the PS drug and 4 involved combination SHA regimens. The most frequent single-agent SHA PS drugs were apalutamide (n = 4) and nilutamide (n = 2), followed by bicalutamide, leuprorelin, fulvestrant, medroxyprogesterone, and anastrozole (n = 1 each). Combination SHA PS regimens were bicalutamide plus leuprorelin (n = 2), flutamide plus leuprorelin (n = 1), and nilutamide plus leuprorelin (n = 1). Fatal outcomes occurred in 4 of these 15 cases. Concomitant SHA exposure was reported in 24 published cases overall. After excluding 3 cases in which the PS regimen itself involved an SHA, 21 cases remained in which an SHA appeared only as concomitant therapy. The most frequent concomitant SHAs were tamoxifen (n = 8), letrozole (n = 7), fulvestrant (n = 7), and exemestane (n = 3), followed by medroxyprogesterone and anastrozole (n = 2 each), and abiraterone, goserelin, toremifene, and enzalutamide (n = 1 each). These patterns provided descriptive clinical context for the concomitant SHA analysis in Model B. (**Table 2**) Detailed case-level information is provided in **Supplementary Table 6**.

**Table 2:** Summary of published interstitial lung disease cases involving sex hormone-pathway agents.

Category	Cases, n	Predominant cancer type(s)	Most frequent SHA(s)/regimen(s)	Fatal cases, n
*SHA in PS regimen, overall*	15	13 prostate; 2 breast	apalutamide (n = 4); nilutamide (n = 2); bicalutamide + leuprorelin (n = 2)	4
*Single-agent SHA as PS*	11	9 prostate; 2 breast	apalutamide (n = 4); nilutamide (n = 2); bicalutamide, leuprorelin, fulvestrant, medroxyprogesterone, and anastrozole (n = 1 each)	2
*Combination SHA PS regimens*	4	4 prostate	bicalutamide + leuprorelin (n = 2); flutamide + leuprorelin (n = 1); nilutamide + leuprorelin (n = 1)	2
*Concomitant SHA exposure, overall*	24	18 breast; 5 prostate; 1 breast + ovarian	tamoxifen (n = 8); letrozole (n = 7); fulvestrant (n = 7); exemestane (n = 3)	3
*Concomitant-only SHA exposure*	21	18 breast; 2 prostate; 1 breast + ovarian	tamoxifen (n = 8); letrozole (n = 7); fulvestrant (n = 7); exemestane (n = 3)	3

Notes: Cases were summarized using standardized drug names and the manuscript-defined SHA classification. PS combination regimens were counted as recorded. Concomitant SHA exposure overall included cases with and without an SHA in the PS regimen, whereas concomitant-only SHA exposure excluded cases in which the PS regimen itself involved an SHA. Frequencies of specific concomitant SHAs may exceed the number of cases because more than one concomitant SHA could be reported in a single case. Abbreviations: ILD, interstitial lung disease; PS, primary suspect; SHA, sex hormone-pathway agent.

## Discussion

Using the FAERS, we provide the first analysis of ILD reporting signals associated with therapies used for sex hormone–sensitive solid tumors in a cross-drug and cross-pathway setting. Focusing on the three hormone-driven malignancies—breast, ovarian, and prostate cancers—we systematically evaluated real-world ILD associations for 65 anticancer agents. Unlike prior work limited to a single drug or tumor type, our analysis focuses on the sex-hormone axis and integrates endocrine therapy, targeted therapy, and chemotherapy, thereby enabling comparisons across therapeutic classes. Because sex hormone-pathway agents (SHAs) are frequently coadministered with newer targeted agents and conventional chemotherapies in clinical practice, we introduced a concomitant SHA model (Model B) to test whether SHAs show independent associations with ILD when used alongside other antineoplastic agents. Notably, even after excluding reports in which an SHA was labeled as the PS, SHAs remained significantly associated with ILD, reducing reliance on reporter attribution and strengthening the robustness of the inference. Methodologically, we combined disproportionality analysis, multivariable logistic regression, multivariable Cox proportional hazards modeling, nonparametric distribution comparisons (ECDF curves with Wilcoxon rank-sum tests), and Weibull TTO analysis to enhance both reliability and clinical utility. Overall, this study addresses an important evidence gap by providing a broad pharmacovigilance overview of ILD reporting associations for antineoplastic agents used in sex hormone–sensitive solid tumors. Although these findings should not be interpreted as absolute risk estimates or comparative clinical risk rankings, they may help prioritize safety signals, support heightened clinical vigilance for selected agents, and guide future studies designed to refine ILD monitoring strategies. To further contextualize these pharmacovigilance findings, we also synthesized published human case reports and case series involving the prespecified agents to provide external clinical context at the case level.

Within SHAs, we observed heterogeneous ILD reporting patterns across therapeutic classes. GnRH agonists and ARPIs showed weak class-level signals (GnRH agonists: ROR = 0.36; 95% CI: 0.33–0.40; ARPIs: ROR = 0.45; 95% CI: 0.41–0.49), whereas ERPAs showed a higher signal (ROR = 1.21; 95% CI: 1.12–1.30). However, class averages masked agent-level heterogeneity. Among GnRH agonists, goserelin showed an elevated signal (ROR = 1.79; 95% CI: 1.47–2.18), consistent with prior pharmacovigilance findings (24), and in Model B, leuprorelin was independently associated with ILD reporting (aOR = 1.69; *P <* 0.001). Among ARPIs, first-generation nonsteroidal antiandrogens flutamide and bicalutamide showed strong signals (flutamide: ROR = 5.81; 95% CI: 3.12–10.83; bicalutamide: ROR = 3.13; 95% CI: 2.60–3.77), consistent with ILD noted in product labeling (25, 26). For newer SHAs (e.g., darolutamide and enzalutamide), ILD has been less emphasized in trials/labeling, yet our analysis identified significant associations. Because darolutamide is often combined with MTIs such as docetaxel, which has an established ILD risk (27), we assessed concomitant exposure: in Model B, darolutamide remained independently associated with ILD reporting even alongside chemotherapy (aOR = 11.56; *P <* 0.001), suggesting potential masking by concomitant drugs. By excluding SHA-primary-suspect reports and focusing on concomitant SHA use, Model B helps unmask potentially underrecognized SHA-related ILD signals. Within ERPAs, Model B identified exemestane as independently associated with ILD reporting (aOR = 1.34; *P =* 0.019), aligning with pathologically confirmed reports of recurrent ILD during exemestane therapy (28). Although pneumonitis/ILD events reported with fulvestrant plus CDK4/6 inhibitors have often been attributed to CDK4/6 inhibitors (29, 30), fulvestrant itself was independently associated with ILD reporting (aOR = 1.45; *P =* 0.001). Toremifene (aOR = 8.35; *P <* 0.001) and medroxyprogesterone (aOR = 3.54; *P <* 0.001) were also independently associated. Taken together, prior research suggested potential ILD signals for nilutamide, flutamide, bicalutamide, goserelin, and apalutamide (24). Building on prior evidence, our study expands the range of SHAs that may warrant attention for potential ILD reporting associations, extending it from previously reported agents such as nilutamide, flutamide, bicalutamide, goserelin, and apalutamide to newer or less-emphasized agents, including darolutamide, fulvestrant, medroxyprogesterone, and toremifene. These findings support heightened pharmacovigilance attention to selected SHAs and suggest that clinicians should remain alert to possible ILD presentations during treatment.

Among nonsex hormone-targeted agents and chemotherapies, multiple classes showed ILD reporting signals. ADCs had the highest class-level signal (ROR = 5.34; 95% CI: 4.98–5.72). The association between T-DXd and ILD was the strongest among individual agents (Model A: aOR = 24.42; *P <* 0.001), which is consistent with the findings of a meta-analysis of clinical data from 1,970 patients with metastatic breast cancer (18) and with the FDA boxed warning (31). Mirvetuximab soravtansine, which targets folate receptor-alpha (FR-α), was also significantly associated with ILD (ROR = 3.37; 95% CI: 2.12–5.36), thereby aligning with reports citing an incidence of ILD/pneumonitis of approximately 10% (32). With respect to mTOR inhibitors, everolimus showed a positive ILD reporting signal (ROR = 4.69; 95% CI: 4.29–5.12). This finding is supported by prior clinical evidence indicating that noninfectious pneumonitis occurs in a subset of patients treated with everolimus, with one prospective study reporting an incidence of approximately 14% (33). Among the PARP inhibitors, olaparib exhibited a stronger ILD reporting signal (ROR = 2.52; 95% CI: 2.27–2.81), consistent with the findings of a study by Ma et al., who reported a significant association between olaparib and ILD (34). ICIs (e.g., pembrolizumab and nivolumab) were also associated with ILD (class ROR = 2.58; 95% CI: 2.32–2.88), which is in line with previous research (35). Among conventional chemotherapies, MTIs showed ILD reporting signals comparable to that in earlier reports (class ROR = 1.53; 95% CI: 1.43–1.62) (36). ANTs (class ROR = 1.80; 95% CI: 1.60–2.02) also showed ILD reporting signals. Collectively, these findings suggest that ILD reporting signals were observed across several pharmacologic classes used for sex hormone–sensitive solid tumors, supporting continued clinical awareness of potential drug-associated pulmonary toxicity. In clinical practice—whether or not drugs are combined—clinicians should maintain heightened vigilance for drug-related ILD, implement routine monitoring for early detection, and initiate timely management to improve quality of life and outcomes.

In addition to the FAERS pharmacovigilance analyses, we systematically reviewed published case reports and case series involving the prespecified agents. This literature-based component was not designed to estimate relative risk, but to provide external case-level evidence that antineoplastic agent-associated ILD has been repeatedly recognized in routine clinical practice. Published cases clustered mainly around agents that were either prominent in our FAERS signal landscape or clinically recognized for pulmonary toxicity, including docetaxel, paclitaxel, trastuzumab, cyclophosphamide, and gemcitabine, thereby providing real-world clinical corroboration for the drug–ILD associations highlighted by our pharmacovigilance analyses. Although fewer reports identified sex hormone-pathway agents (SHAs) as the primary suspect regimen, SHAs were repeatedly documented as concomitant therapies in ILD cases. This pattern provides a clinically grounded rationale for Model B and supports evaluating SHA exposure beyond reporter-designated primary-suspect attribution alone. Overall, the literature synthesis strengthens the clinical interpretability of our findings while underscoring that FAERS captures a broader SHA-related reporting-signal landscape than is represented in the published case literature.

Beyond drug-level clinical context, the published cases also helped contextualize the reported timing of ILD. In our review, ILD was often described within the first few months after treatment initiation, and fatal outcomes were reported in some cases. Consistently, in the FAERS complete-case exploratory TTO analysis, death-recorded reports had shorter reported TTO than non–death-recorded reports (median 56 vs. 66 days), highlighting the importance of early recognition and timely clinical evaluation. Lower body weight was associated with higher odds of ILD reporting in the multivariable logistic regression model and with shorter reported TTO in the exploratory Cox model. In exploratory body-weight subgroup analyses, no significant TTO difference between death-recorded and non–death-recorded reports was observed in the <70 kg subgroup after FDR correction, whereas death-recorded reports had shorter reported TTO than non–death-recorded reports in the ≥70 kg subgroup. This divergent pattern suggests that body weight may act as a potential modifier of reported onset–fatality patterns in FAERS. However, because FAERS lacks exposure denominators, systematic follow-up, complete treatment-duration information, and confirmed causes of death, these findings should not be interpreted as conventional risk or survival estimates. Overall, these TTO and body-weight findings are hypothesis-generating and require validation in prospective or real-world studies before they can be used to refine clinical monitoring strategies.

These findings have important clinical implications for the safety management of patients with sex hormone–sensitive solid tumors. Because ILD reporting signals were observed across multiple therapeutic classes, clinicians should maintain a threshold for considering drug-associated ILD when patients develop new or unexplained respiratory symptoms, hypoxemia, ground-glass opacities, interstitial changes on imaging, or pulmonary findings that cannot be readily explained by infection, tumor progression, radiation injury, pulmonary embolism, or cardiac disease. Rather than serving as a clinical risk-ranking tool, our results may help prioritize safety vigilance for agents with stronger or clinically recognized ILD reporting associations, such as T-DXd, selected CDK4/6 inhibitors, mTOR inhibitors, ICIs, PARP inhibitors, taxanes, and selected SHAs. For such agents, informed consent and patient education should include discussion of possible ILD/pneumonitis symptoms and the need to report dyspnea, cough, fever, or oxygen desaturation promptly. Baseline chest imaging and pulmonary assessment may be considered when clinically appropriate, particularly in patients with pre-existing lung disease, prior thoracic radiotherapy, respiratory symptoms, or planned treatment with agents known or suspected to have pulmonary toxicity; subsequent reassessment should be guided by symptoms, oxygenation, imaging findings, treatment phase, and the suspected agent. When drug-associated ILD is suspected, timely interruption of the suspected therapy, exclusion of alternative causes, early chest imaging, severity assessment, and multidisciplinary management involving oncology and pulmonology teams are essential. Treatment decisions, including corticosteroid use and drug rechallenge or discontinuation, should follow existing clinical guidance, event severity, and the individual therapeutic context. Thus, the main clinical value of this study is to support earlier recognition, structured evaluation, and signal-informed vigilance rather than to prescribe a uniform surveillance pathway for all patients. These translational implications are particularly relevant in the contemporary management of prostate cancer, where recent studies have emphasized marked tumor heterogeneity and the need for personalized therapeutic strategies (37). Recent evidence has also highlighted androgen receptor-pathway dependence, intratumoral androgen biology, genetic variation, and resistance to novel hormonal therapies as key factors shaping treatment selection and sequencing (38, 39). In this context, treatment optimization should integrate not only antitumor efficacy and resistance mechanisms, but also patient-level susceptibility and drug-specific safety signals. Therefore, our findings may complement emerging precision-oncology frameworks by adding a pharmacovigilance-based safety perspective to individualized treatment planning and monitoring for sex hormone-sensitive solid tumors.

This study has several limitations. First, FAERS is a spontaneous reporting system; therefore, underreporting, selective or stimulated reporting, incomplete information, and variable data quality are inherent. Because exposure denominators and systematic follow-up are unavailable, RORs and regression-derived aORs cannot estimate incidence, absolute risk, causal effects, or comparative clinical risk, and should instead be interpreted as reporting associations for signal detection. Second, although the reporting-year-adjusted sensitivity analysis supported the robustness of the major drug-level associations, residual reporting bias related to time on market, regulatory attention, and differential clinical surveillance cannot be fully excluded. Third, important clinical variables, including comorbidities, baseline pulmonary disease, prior thoracic radiotherapy, treatment line, dosing, treatment sequence, and treatment duration, were incompletely recorded, leaving potential residual confounding despite multivariable adjustment. Fourth, TTO analyses were exploratory complete-case analyses because therapy start dates and event onset dates are not mandatory in FAERS. Accordingly, Cox, Weibull, and subgroup TTO analyses should be interpreted as descriptive assessments of reported onset patterns rather than conventional survival analyses. Fifth, the FAERS death outcome denotes death recorded in the adverse-event report, not adjudicated ILD-attributable death. Therefore, further prospective real-world studies are needed to validate these FAERS-derived signals and better quantify the clinical risk and patient-level determinants of antineoplastic agent-associated ILD.

In summary, this multicancer FAERS study provides a broad pharmacovigilance overview of ILD reporting associations among antineoplastic agents used for breast, ovarian, and prostate cancers. Our findings confirm prominent reporting signals for several therapies already recognized for pulmonary toxicity and further highlight underrecognized signals for selected sex hormone-pathway agents, particularly in the context of concomitant treatment. Beyond drug-level signals, patient factors also appeared relevant: older age and lower body weight were associated with higher odds of ILD reporting, and body weight may further influence the reported relationship between onset timing and fatal outcomes. Together, these findings support a more integrated approach to ILD vigilance that considers not only the suspected drug and therapeutic class, but also concomitant endocrine-pathway exposure and patient-level susceptibility. Because FAERS data cannot establish incidence, causality, or comparative clinical risk, these results should be interpreted as hypothesis-generating reporting signals and validated in prospective or real-world studies with confirmed drug exposure, imaging-based ILD diagnosis, and well-characterized clinical outcomes.

## Data Availability

Publicly available datasets were analyzed in this study. This data can be found here: https://fis.fda.gov/extensions/FPD-QDE-FAERS/FPD-QDE-FAERS.html.
