# Supplementary material for: Antineoplastic agent-associated interstitial lung disease in breast, ovarian, and prostate cancers: a pharmacovigilance study using the FDA adverse event reporting system
Source: Front Immunol. 2026 Jun 10;17:1840323. doi: 10.3389/fimmu.2026.1840323 (PMC13290764; doi:10.3389/fimmu.2026.1840323)
Supplement: Supplementary file 1 [file DataSheet1.pdf]

Supplementary Material

Material and methods

Data source

Details of data cleaning and deduplication are as follows. All the datasets were imported into PostgreSQL for integrated analyses. To remove duplicates, we followed FDA recommendations: for records sharing the same CASEID, we retained the entry with the most recent FDA_DT; for cases where the CASEID and the FDA_DT were identical, we retained the record with the larger PRIMARYID. After this initial deduplication, we still identified a small number of duplicate PRIMARYID values and therefore performed a second pass to ensure that one unique report was in the analytic database.

Table S1: **Preferred terms included in the narrow-scope MedDRA SMQ for interstitial lung disease**

code	PT
10001881	Alveolar proteinosis
10001889	Alveolitis
10006448	Bronchiolitis
10014952	Eosinophilia myalgia syndrome
10014962	Eosinophilic pneumonia
10021240	Idiopathic pulmonary fibrosis
10022611	Interstitial lung disease
10025102	Lung infiltration
10029888	Obliterative bronchiolitis
10035742	Pneumonitis
10036805	Progressive massive fibrosis
10037383	Pulmonary fibrosis
10037457	Pulmonary vasculitis
10037754	Radiation alveolitis
10037758	Radiation fibrosis - lung
10037765	Radiation pneumonitis
10050343	Alveolitis necrotising
10052235	Transfusion-related acute lung injury
10052832	Eosinophilic pneumonia acute
10052833	Eosinophilic pneumonia chronic
10058824	Pulmonary necrosis
10060902	Diffuse alveolar damage
10061473	Pulmonary radiation injury
10061924	Pulmonary toxicity
10063725	Idiopathic pneumonia syndrome
10066728	Acute interstitial pneumonitis

10070831	Necrotising bronchiolitis
10073344	Alveolar lung disease
10076515	Combined pulmonary fibrosis and emphysema
10078117	Eosinophilic granulomatosis with polyangiitis
10078268	Idiopathic interstitial pneumonia
10080547	Small airways disease
10080701	Autoimmune lung disease
10081792	Lung opacity
10081988	Hypersensitivity pneumonitis
10083303	Bronchiolitis obliterans syndrome
10084305	Pleuroparenchymal fibroelastosis
10085188	Probable e-cigarette or vaping product use associated lung injury
10085189	Confirmed e-cigarette or vaping product use associated lung injury
10085352	Immune-mediated lung disease
10085517	Rheumatoid arthritis-associated interstitial lung disease
10085628	Radiation bronchitis
10086041	Chronic graft versus host disease in lung
10086117	Low lung compliance
10087834	Interstitial lung abnormality

Abbreviations: ILD, interstitial lung disease; MedDRA, Medical Dictionary for Regulatory Activities; PT, preferred term; SMQ, Standardized MedDRA Query.

Statistical analysis

Table S2: **2×2 contingency table for calculation of the ROR**

	Target adverse events	Other adverse events
Target drugs	a	b
Other drugs	c	d

Notes: a = reports with index drug and ILD; b = reports with index drug and other adverse events; c = reports with other drugs and ILD; d = reports with other drugs and other adverse events. ROR = (a/c)/(b/d) = ad/bc; 95% CI = exp{ln(ROR) ± 1.96 × √(1/a + 1/b + 1/c + 1/d)}. Abbreviations: ILD = interstitial lung disease; ROR = reporting odds ratio; CI = confidence interval.

To define a neutral comparator for the comparative models, we excluded sex hormone-pathway agents (SHAs) and agents classified as novel targeted modalities. Among the remaining candidates, we identified drugs with a disproportionality lower bound ≤ 1.0 (ROR_L ≤ 1) and without explicit ILD warnings in labels or published evidence. From these, the agent with the largest case count in the filtered cohort was selected; this procedure yielded capecitabine as the reference drug.

After selecting capecitabine as the reference drug and conducting the primary Model A and Model B analyses, we performed an additional sensitivity analysis of Model A by including reporting year as a categorical covariate. Drug-level estimates from the primary Model A and the reporting-year-adjusted Model A were compared in terms of adjusted odds ratios (aORs), 95% CIs, effect direction, and whether the lower bound of the 95% CI remained >1.

In addition to drug-level reporting associations, we performed exploratory analyses of reported onset timing among ILD reports with available date information. Time-to-onset (TTO) was calculated as the interval from therapy start date to ILD event onset date. Complete-case TTO analyses included ILD reports with both dates available and a non-negative calculated TTO; reports with missing therapy start dates, missing event onset dates, or negative TTO values were excluded. To assess potential date-completeness bias, we compared TTO-included and TTO-excluded reports with respect to demographic, tumor-related, drug-related, reporting-period, and outcome-related characteristics. Continuous variables were summarized as median (interquartile range [IQR]) and compared using the Wilcoxon rank-sum test or t test, as appropriate. Categorical variables were summarized as n (%) and compared using Pearson chi-square or Fisher exact tests. Standardized mean differences (SMDs) were also calculated to quantify between-group imbalance; an absolute SMD >0.10 was interpreted as suggesting meaningful imbalance.

Separately, we summarized death outcomes recorded in FAERS among ILD reports. Death was identified when the FAERS OUTC field contained a death record. All other ILD reports were classified as non–death-recorded ILD reports. This classification reflects the outcome information reported to FAERS and does not establish that ILD was the confirmed cause of death. For each drug or pharmacologic class with ≥10 death-recorded ILD reports, the proportion of ILD reports with death recorded was calculated as the number of death-recorded ILD reports divided by the total number of ILD reports for that drug or class.

Among complete-case TTO reports, differences in reported TTO between death-recorded and non–death-recorded ILD reports were described using empirical cumulative distribution functions (ECDFs) and compared using Wilcoxon rank-sum tests. Stratified summaries were performed by body weight (<70 kg vs. ≥70 kg) and by age category. These subgroup analyses were exploratory. The 70-kg cutoff was considered clinically interpretable because it approximates the average adult reference body weight used in FDA oncology dose-adjustment guidance and related pharmacologic calculations[1]; it was not considered an ILD-specific clinical risk threshold. Nominal subgroup *P* values were adjusted using the Benjamini–Hochberg false discovery rate (FDR) method.

We additionally fitted multivariable Cox proportional hazards models including age, weight, and the number of concomitant medications. To characterize reported TTO distributions for each drug–ILD pair, two-parameter Weibull models were fitted when the pair had ≥3 nonmissing TTO values with ≥2 distinct values. Ninety-five percent confidence intervals (CIs) for the Weibull shape parameter β and scale parameter α were computed on the log scale and back-transformed. Reported onset patterns were classified by the 95% CI for β as “early” if the upper bound was <1, “wear-out” if the lower bound was >1, and “random” otherwise.

Results

Disproportionality analysis

Drugs	Cases	ROR (95% CI)
Leuprorelin	217	0.40 (0.35–0.45)
Goserelin	93	2.09 (1.70–2.58)
Triptorelin	3	0.13 (0.04–0.40)
Enzalutamide	86	0.30 (0.24–0.37)
Abiraterone	119	0.38 (0.32–0.46)
Bicalutamide	95	2.34 (1.90–2.88)
Apalutamide	92	1.24 (1.00–1.52)
Darolutamide	38	1.64 (1.18–2.26)
Flutamide	11	5.30 (2.81–9.97)
Nilutamide	8	15.40 (6.74–35.20)
Letrozole	279	1.07 (0.94–1.20)
Anastrozole	118	1.00 (0.83–1.20)
Fulvestrant	126	1.03 (0.86–1.23)
Exemestane	122	1.66 (1.38–1.99)
Tamoxifen	22	0.38 (0.25–0.58)
Toremifene	2	0.66 (0.16–2.69)
Medroxyprogesterone	2	1.38 (0.34–5.66)
Palbociclib	235	0.38 (0.33–0.43)
Abemaciclib	130	1.65 (1.38–1.97)
Ribociclib	153	1.08 (0.92–1.27)
Trastuzumab Deruxtecan	702	8.13 (7.47–8.84)
Trastuzumab Emtansine	142	1.86 (1.57–2.21)
Sacituzumab Govitecan	42	0.72 (0.53–0.97)
Mirvetuximab Soravtansine	14	2.94 (1.71–5.08)
Trastuzumab	569	1.09 (1.00–1.19)
Pertuzumab	227	1.80 (1.57–2.06)
Bevacizumab	144	0.59 (0.50–0.70)
Pertuzumab Trastuzumab Vorhyaluronidase Alfa	7	0.13 (0.06–0.27)
Pertuzumab Trastuzumab	1	0.69 (0.10–4.99)
Denosumab	21	0.21 (0.14–0.32)
Lapatinib	58	0.61 (0.47–0.80)
Tucatinib	11	0.31 (0.17–0.56)
Neratinib	2	0.06 (0.02–0.25)
Olaparib	277	2.69 (2.37–3.04)
Niraparib	87	0.64 (0.51–0.79)
Rucaparib	3	0.03 (0.01–0.08)
Talazoparib	5	0.47 (0.19–1.13)
Pembrolizumab	140	1.61 (1.36–1.91)
Atezolizumab	140	2.41 (2.03–2.87)
Nivolumab	26	1.12 (0.76–1.66)
Ipilimumab	12	2.16 (1.21–3.87)
Durvalumab	3	0.94 (0.30–2.95)
Toripalimab	1	6.09 (0.73–50.60)
Everolimus	460	4.14 (3.75–4.57)
Alpelisib	27	0.52 (0.36–0.76)
Capivasertib	5	0.82 (0.34–1.98)
Paclitaxel	446	1.44 (1.31–1.59)
Docetaxel	436	1.90 (1.72–2.09)
Eribulin	168	2.26 (1.93–2.65)
Cabazitaxel	49	1.54 (1.16–2.05)
Vinorelbine	10	0.86 (0.46–1.62)
Estramustine	9	1.61 (0.82–3.13)
Capecitabine	94	0.48 (0.39–0.59)
Gemcitabine	72	1.43 (1.13–1.81)
Fluorouracil	36	1.42 (1.01–1.98)
Topotecan	10	0.87 (0.46–1.63)
Etoposide	7	0.82 (0.39–1.74)
Irinotecan	6	1.91 (0.84–4.34)
Carboplatin	84	0.36 (0.29–0.44)
Cisplatin	9	0.57 (0.29–1.09)
Doxorubicin	198	1.46 (1.27–1.69)
Epirubicin	91	1.03 (0.84–1.27)
Cyclophosphamide	63	0.43 (0.33–0.55)
Zoledronic Acid	34	0.30 (0.21–0.42)
Lenalidomide	5	0.52 (0.21–1.25)

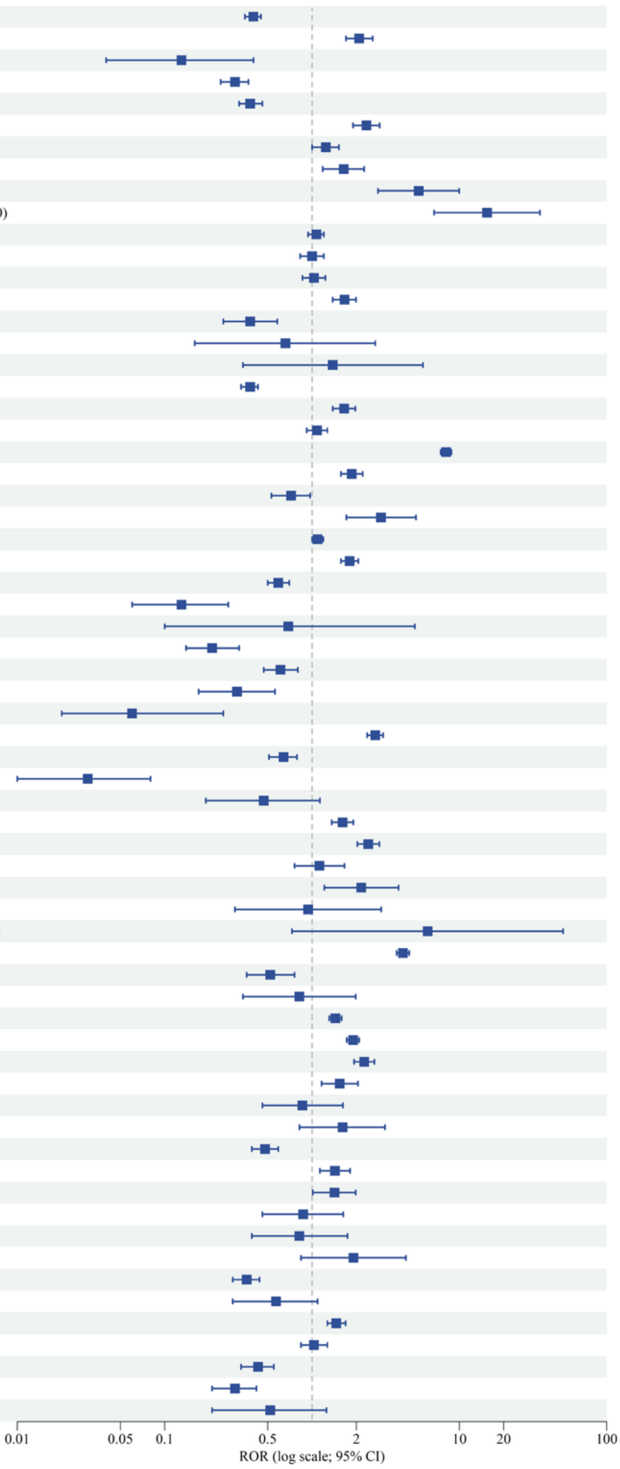

Figure S1: **Sensitivity analysis (HCP-only reporters): drug-level RORs for ILD in sex hormone–sensitive solid tumors.** Notes: We restricted reporters to HCP in FAERS and recomputed ROR-based disproportionality by drug; the forest plot showed RORs with 95% CIs on a log scale. A positive reporting signal was defined as a lower 95% CI bound > 1 with ≥4 co-reports.

Abbreviations: ROR, reporting odds ratio; CI, confidence interval; HCP, healthcare professional; ILD, interstitial lung disease; FAERS, FDA Adverse Event Reporting System.

Drugs	Cases	ROR (95% CI)
Leuprorelin	207	0.45 (0.39–0.52)
Goserelin	84	3.05 (2.45–3.79)
Enzalutamide	93	0.28 (0.23–0.34)
Abiraterone	68	0.48 (0.38–0.61)
Bicalutamide	84	4.59 (3.69–5.73)
Apalutamide	66	1.87 (1.46–2.39)
Darolutamide	25	1.89 (1.27–2.81)
Flutamide	10	10.99 (5.74–21.05)
Nilutamide	6	21.42 (8.95–51.26)
Letrozole	159	1.38 (1.18–1.62)
Anastrozole	94	1.23 (1.00–1.51)
Fulvestrant	94	1.63 (1.33–2.01)
Exemestane	56	1.31 (1.00–1.71)
Tamoxifen	6	0.20 (0.09–0.44)
Toremifene	9	4.25 (2.18–8.27)
Medroxyprogesterone	1	1.39 (0.19–10.00)
Palbociclib	96	0.23 (0.19–0.29)
Abemaciclib	175	2.43 (2.08–2.83)
Ribociclib	50	0.49 (0.37–0.65)
Trastuzumab Deruxtecan	495	13.50 (12.23–14.89)
Trastuzumab Emtansine	60	1.86 (1.44–2.41)
Sacituzumab Govitecan	9	0.40 (0.21–0.77)
Mirvetuximab Soravtansine	1	0.35 (0.05–2.50)
Trastuzumab	260	1.26 (1.11–1.43)
Pertuzumab	148	3.03 (2.57–3.58)
Bevacizumab	82	0.91 (0.73–1.14)
Pertuzumab Trastuzumab Vorhyaluronidase Alfa	2	0.08 (0.02–0.33)
Denosumab	11	0.27 (0.15–0.49)
Lapatinib	33	0.53 (0.37–0.74)
Tucatinib	1	0.06 (0.01–0.40)
Olaparib	257	3.78 (3.32–4.29)
Niraparib	54	0.40 (0.31–0.52)
Talazoparib	2	0.44 (0.11–1.75)
Pembrolizumab	43	1.22 (0.90–1.65)
Atezolizumab	67	3.28 (2.57–4.19)
Nivolumab	1	0.10 (0.01–0.73)
Ipilimumab	1	0.42 (0.06–3.02)
Toripalimab	1	3.36 (0.46–24.52)
Everolimus	127	2.01 (1.68–2.41)
Alpelisib	6	0.20 (0.09–0.44)
Capivasertib	3	0.69 (0.22–2.14)
Paclitaxel	148	1.29 (1.09–1.52)
Docetaxel	221	0.91 (0.80–1.04)
Eribulin	140	5.47 (4.60–6.50)
Cabazitaxel	34	2.94 (2.09–4.14)
Vinorelbine	6	1.01 (0.45–2.25)
Estramustine	9	4.23 (2.17–8.24)
Capecitabine	31	0.31 (0.21–0.44)
Gemcitabine	20	0.95 (0.61–1.48)
Fluorouracil	26	2.68 (1.81–3.96)
Topotecan	6	1.40 (0.63–3.13)
Etoposide	4	1.23 (0.46–3.29)
Irinotecan	6	5.11 (2.26–11.59)
Carboplatin	29	0.35 (0.24–0.51)
Cisplatin	4	0.73 (0.27–1.95)
Doxorubicin	92	1.86 (1.51–2.29)
Epirubicin	69	2.11 (1.66–2.69)
Cyclophosphamide	21	0.39 (0.25–0.59)
Zoledronic Acid	14	0.26 (0.15–0.43)

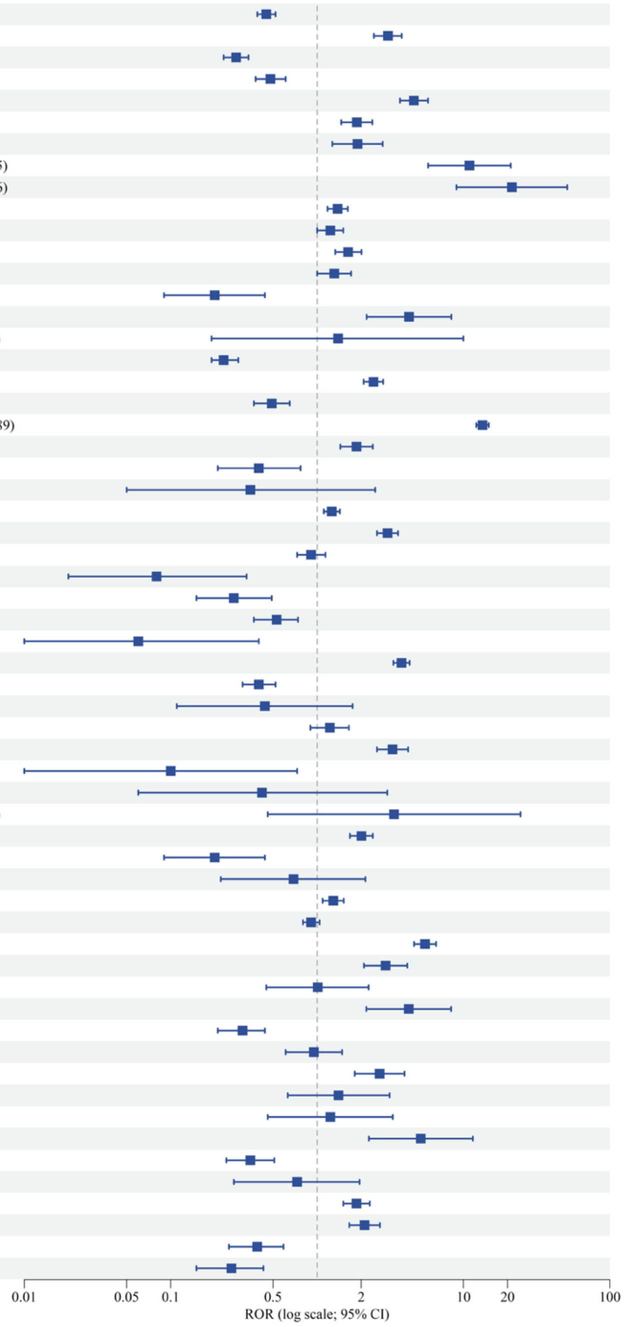

Figure S2: **Sensitivity analysis (single-PT case definition): drug-level RORs for ILD in sex hormone-sensitive solid tumors.** Notes: We restricted cases to the single MedDRA PT “Interstitial lung disease” and recomputed drug-level ROR-based disproportionality; the forest plot displayed RORs with 95% CIs on a log scale. A positive reporting signal was defined as a lower 95% CI bound > 1 with ≥4 co-reports. Abbreviations: ROR, reporting odds ratio; CI, confidence interval; PT, preferred term; ILD, interstitial lung disease; MedDRA, Medical Dictionary for Regulatory Activities.

To test the robustness of the main analysis, we conducted two sensitivity analyses. First, we restricted reporters to healthcare professionals as operationalized in the FAERS and recomputed ROR-based disproportionality metrics. (Figure S1) Second, we applied a stricter case definition that included only

the MedDRA preferred term “Interstitial lung disease” as the sole PT for case inclusion. (Figure S2) In both analyses, the directions of the reporting signals and their statistical significance were broadly consistent with the main analysis across all drugs and classes, indicating that our conclusions were not driven by potential differential reporting from nonprofessional reporters and did not depend on using a broader versus narrower PT set.

Table S3: **Sensitivity analysis of Model A additionally adjusted for reporting year**

Drug	Primary Model A aOR (95% CI)	Primary *P* value	Reporting-year-adjusted Model A aOR (95% CI)	Adjusted *P* value	Direction retained	Signal retained after reporting-year adjustment
Leuprorelin	2.52 (1.55–4.08)	<0.001	2.43 (1.49–3.94)	<0.001	Yes	Yes
Goserelin	6.99 (3.90–12.53)	<0.001	5.66 (3.14–10.20)	<0.001	Yes	Yes
Triptorelin	0.00 (0.00–NA)	0.978	0.00 (0.00–NA)	0.978	Yes	No signal in either model
Enzalutamide	1.87 (1.05–3.32)	0.033	1.86 (1.04–3.31)	0.036	Yes	Yes
Abiraterone	1.40 (0.84–2.33)	0.196	1.42 (0.85–2.37)	0.177	Yes	No signal in either model
Bicalutamide	6.37 (3.73–10.87)	<0.001	5.69 (3.33–9.74)	<0.001	Yes	Yes
Apalutamide	4.48 (2.67–7.51)	<0.001	5.03 (2.99–8.46)	<0.001	Yes	Yes
Darolutamide	8.76 (3.91–19.67)	<0.001	8.48 (3.76–19.16)	<0.001	Yes	Yes
Flutamide	8.06 (1.00–65.23)	0.05	5.96 (0.73–48.47)	0.095	Yes	No signal in either model
Nilutamide	17.82 (3.67–86.62)	<0.001	15.38 (3.15–75.09)	<0.001	Yes	Yes

Letrozole	4.48 (2.87–6.99)	<0.001	4.64 (2.97–7.25)	<0.001	Yes	Yes
Anastrozole	1.82 (1.08–3.07)	0.024	1.59 (0.94–2.69)	0.084	Yes	Attenuated/ not retained
Fulvestrant	2.88 (1.72–4.83)	<0.001	2.91 (1.74–4.88)	<0.001	Yes	Yes
Exemestane	2.37 (1.38–4.08)	0.002	2.12 (1.23–3.65)	0.007	Yes	Yes
Tamoxifen	1.50 (0.71–3.18)	0.286	1.24 (0.58–2.64)	0.573	Yes	No signal in either model
Toremifene	6.69 (0.86–52.14)	0.069	5.00 (0.63–39.56)	0.127	Yes	No signal in either model
Medroxyprogesterone	0.00 (0.00–NA)	0.992	0.00 (0.00–NA)	0.992	Yes	No signal in either model
Palbociclib	1.81 (1.16–2.82)	0.009	1.94 (1.24–3.03)	0.003	Yes	Yes
Abemaciclib	19.87 (12.56–31.45)	<0.001	21.88 (13.78–34.74)	<0.001	Yes	Yes
Ribociclib	3.41 (2.08–5.57)	<0.001	3.79 (2.31–6.20)	<0.001	Yes	Yes
Trastuzumab Deruxtecan	24.42 (15.70–38.00)	<0.001	27.28 (17.41–42.75)	<0.001	Yes	Yes
Trastuzumab Emtansine	5.01 (3.08–8.16)	<0.001	4.91 (3.00–8.02)	<0.001	Yes	Yes
Sacituzumab Govitecan	3.55 (1.83–6.88)	<0.001	3.86 (1.98–7.51)	<0.001	Yes	Yes
Mirvetuximab Soravtansine	11.86 (3.31–42.43)	<0.001	11.44 (3.19–41.08)	<0.001	Yes	Yes

Trastuzumab	2.84 (1.82–4.42)	<0.001	2.88 (1.85–4.49)	<0.001	Yes	Yes
Pertuzumab	4.76 (2.99–7.56)	<0.001	4.86 (3.05–7.75)	<0.001	Yes	Yes
Bevacizumab	1.44 (0.88–2.36)	0.148	1.37 (0.83–2.24)	0.216	Yes	No signal in either model
Pertuzumab Trastuzumab Vorhyaluronidase Alfa	1.58 (0.37–6.79)	0.539	1.66 (0.38–7.17)	0.497	Yes	No signal in either model
Pertuzumab Trastuzumab	2.85 (0.37–21.93)	0.315	3.26 (0.42–25.13)	0.257	Yes	No signal in either model
Denosumab	1.01 (0.43–2.37)	0.983	0.98 (0.42–2.30)	0.958	No	No signal in either model
Lapatinib	1.95 (1.17–3.28)	0.011	1.66 (0.98–2.80)	0.058	Yes	Attenuated/ not retained
Tucatinib	1.75 (0.60–5.12)	0.308	1.95 (0.66–5.71)	0.225	Yes	No signal in either model
Neratinib	3.34 (0.44–25.66)	0.246	3.21 (0.42–24.78)	0.263	Yes	No signal in either model
Olaparib	10.88 (6.85–17.27)	<0.001	11.93 (7.50–18.97)	<0.001	Yes	Yes
Niraparib	1.49 (0.75–2.96)	0.257	1.65 (0.83–3.29)	0.154	Yes	No signal in either model
Rucaparib	0.44 (0.06–3.24)	0.417	0.50 (0.07–3.75)	0.503	Yes	No signal in either model
Talazoparib	1.35 (0.40–4.55)	0.627	1.51 (0.45–5.08)	0.509	Yes	No signal in either model
Pembrolizumab	4.31 (2.42–7.67)	<0.001	4.67 (2.61–8.36)	<0.001	Yes	Yes

Atezolizumab	3.96 (2.45–6.40)	<0.001	4.50 (2.78–7.31)	<0.001	Yes	Yes
Nivolumab	6.22 (3.33–11.61)	<0.001	6.76 (3.61–12.65)	<0.001	Yes	Yes
Ipilimumab	5.33 (1.56–18.27)	0.008	5.52 (1.61–18.96)	0.007	Yes	Yes
Durvalumab	2.79 (0.64–12.18)	0.171	3.13 (0.72–13.67)	0.13	Yes	No signal in either model
Toripalimab	0.00 (0.00–NA)	0.998	0.00 (0.00–NA)	0.998	Yes	No signal in either model
Everolimus	12.84 (8.22–20.05)	<0.001	12.48 (7.97–19.54)	<0.001	Yes	Yes
Alpelisib	1.86 (0.92–3.77)	0.084	2.10 (1.04–4.27)	0.04	Yes	New after adjustment
Capivasertib	5.37 (1.54–18.76)	0.008	5.31 (1.50–18.71)	0.009	Yes	Yes
Paclitaxel	3.68 (2.38–5.70)	<0.001	3.74 (2.41–5.79)	<0.001	Yes	Yes
Docetaxel	3.36 (2.16–5.24)	<0.001	3.47 (2.23–5.41)	<0.001	Yes	Yes
Eribulin	3.95 (2.43–6.43)	<0.001	3.88 (2.38–6.32)	<0.001	Yes	Yes
Cabazitaxel	4.10 (2.37–7.12)	<0.001	3.81 (2.19–6.63)	<0.001	Yes	Yes
Vinorelbine	3.59 (1.05–12.23)	0.041	3.20 (0.94–10.93)	0.064	Yes	Attenuated/ not retained
Estramustine	1.11 (0.15–8.38)	0.918	0.99 (0.13–7.51)	0.995	No	No signal in either model

Capecitabine (REF)	1.00 (Reference)		1.00 (Reference)		REF	REF
Gemcitabine	5.16 (2.94–9.05)	<0.001	4.51 (2.57–7.94)	<0.001	Yes	Yes
Fluorouracil	2.13 (0.73–6.24)	0.168	2.18 (0.74–6.40)	0.155	Yes	No signal in either model
Topotecan	1.92 (0.72–5.13)	0.192	1.62 (0.61–4.35)	0.335	Yes	No signal in either model
Etoposide	0.00 (0.00–NA)	0.987	0.00 (0.00–NA)	0.987	Yes	No signal in either model
Irinotecan	13.59 (4.69–39.36)	<0.001	10.13 (3.42–30.04)	<0.001	Yes	Yes
Carboplatin	0.48 (0.26–0.90)	0.022	0.52 (0.28–0.96)	0.037	Yes	No signal in either model
Cisplatin	0.00 (0.00–NA)	0.979	0.00 (0.00–NA)	0.979	Yes	No signal in either model
Doxorubicin	4.01 (2.45–6.58)	<0.001	3.74 (2.28–6.14)	<0.001	Yes	Yes
Epirubicin	2.09 (1.20–3.65)	0.01	2.08 (1.19–3.64)	0.01	Yes	Yes
Cyclophosphamide	0.76 (0.42–1.40)	0.379	0.81 (0.44–1.48)	0.484	Yes	No signal in either model
Zoledronic Acid	2.03 (0.98–4.21)	0.056	1.80 (0.87–3.73)	0.114	Yes	No signal in either model
Lenalidomide	1.83 (0.42–7.90)	0.417	1.53 (0.35–6.62)	0.571	Yes	No signal in either model

Notes: Primary Model A used capecitabine as the reference drug and adjusted for age, body weight, comedication, and reporter type. In the sensitivity analysis, reporting year was additionally included as a covariate to assess the robustness of drug-level adjusted associations. Values are presented as adjusted odds ratios (aORs) with 95% confidence intervals (CIs). Direction retained indicates whether the direction of the association was consistent between the primary Model A and the reporting-year-

adjusted model. Signal retained after reporting-year adjustment indicates whether the lower bound of the 95% CI remained >1 after additional adjustment for reporting year. Estimates should be interpreted as reporting associations rather than comparative clinical risks. Abbreviations: aOR, adjusted odds ratio; CI, confidence interval.

Analysis of death-recorded reports

Table S4: **Inclusion and exclusion flow for the time-to-onset analysis**

TTO inclusion/exclusion flow		
Flow item	n	% of all ILD reports
Total ILD reports	8146	100
Included in TTO analysis	3464	42.5
Excluded from TTO analysis	4682	57.5
Missing both therapy start and event onset dates	2973	36.5
Missing therapy start date only	911	11.2
Missing event onset date only	609	7.5
Negative TTO	189	2.3

Notes: The source population comprised FAERS reports of interstitial lung disease associated with antineoplastic therapy for sex hormone-sensitive solid tumors. Time to onset (TTO) was calculated as the interval from therapy start date to event onset date. Reports were included in the TTO analysis only when both dates were available and the calculated TTO was ≥0 days. Reports were excluded if both dates were missing, if either therapy start date or event onset date was missing, or if the calculated TTO was negative. Percentages were calculated using all ILD reports as the denominator. Abbreviations: FAERS, FDA Adverse Event Reporting System; ILD, interstitial lung disease; TTO, time to onset.

Table S5: **Baseline characteristics of reports included in and excluded from the time-to-onset analysis**

Characteristics of TTO-included and TTO-excluded reports				
Characteristic	Excluded from TTO analysis	Included in TTO analysis	*P* value	SMD
Number of reports	4682	3464		
Sex			<0.001	0.342
Female	3540 (75.6)	2706 (78.1)		
Male	616 (13.2)	651 (18.8)		
Missing/Other	526 (11.2)	107 (3.1)		
Age, years, median [IQR]	64.00 [53.00, 72.62]	66.00 [56.00, 74.00]	<0.001	0.204
Weight, kg, median [IQR]	67.70 [56.00, 78.00]	62.00 [52.00, 74.81]	<0.001	0.231
Cancer type			<0.001	0.147
Breast cancer	3658 (78.1)	2518 (72.7)		
Ovarian cancer	414 (8.8)	314 (9.1)		
Prostate cancer	610 (13.0)	632 (18.2)		
Drug class			<0.001	0.323

GnRH agonists	196 (4.2)	189 (5.5)		
ARPIs	271 (5.8)	260 (7.5)		
ERPAs	484 (10.3)	323 (9.3)		
CDK4/6 inhibitors	501 (10.7)	364 (10.5)		
ADCs	671 (14.3)	300 (8.7)		
mAbs	653 (13.9)	381 (11.0)		
TKIs	35 (0.7)	57 (1.6)		
PARP inhibitors	279 (6.0)	203 (5.9)		
ICIs	184 (3.9)	172 (5.0)		
PI3K/AKT/mTOR inhibitors	340 (7.3)	270 (7.8)		
MTIs	558 (11.9)	652 (18.8)		
AMs	139 (3.0)	90 (2.6)		
PBC	78 (1.7)	23 (0.7)		
ANTs	193 (4.1)	111 (3.2)		
AAs	43 (0.9)	35 (1.0)		
BPs	45 (1.0)	15 (0.4)		
Other/sparse drug classes	12 (0.3)	19 (0.5)		
Report period			<0.001	0.343
2004–2010	279 (6.0)	507 (14.6)		
2011–2015	641 (13.7)	585 (16.9)		
2016–2020	1382 (29.5)	1042 (30.1)		
2021–2025	2380 (50.8)	1330 (38.4)		
Death	845 (18.0)	851 (24.6)	<0.001	0.16
Life-threatening	236 (5.0)	290 (8.4)	<0.001	0.133
Hospitalization	1237 (26.4)	1499 (43.3)	<0.001	0.359
Other serious	2112 (45.1)	736 (21.2)	<0.001	0.524
Disability	27 (0.6)	34 (1.0)	0.038	0.046
Required intervention	4 (0.1)	11 (0.3)	0.019	0.052

Notes: Values are presented as *n* (%) unless otherwise stated. Continuous variables are summarized as median [interquartile range]. TTO-included reports were defined as reports with available therapy start date and event onset date and a calculated TTO ≥0 days. TTO-excluded reports were defined as reports lacking the required date information or having a negative calculated TTO. *P* values compare TTO-included and TTO-excluded reports. Standardized mean differences (SMDs) were calculated to quantify between-group imbalance; an absolute SMD >0.10 was interpreted as suggesting meaningful imbalance. For multi-category variables, including sex, cancer type, drug class, report period, and serious outcome, *P* values and SMDs refer to the overall distribution; subcategory rows are descriptive only. Percentages for serious outcome categories may not sum to 100% because reports with missing serious outcome information are not displayed. Missing/other sex records are shown as a separate category. Topoisomerase inhibitors and immunomodulatory drugs were merged into "Other/sparse drug classes" in the source output because of sparse counts. Abbreviations: ADCs, antibody–drug conjugates; AAs, alkylating agents; AMs, antimetabolites; ANTs, anthracycline antibiotics; ARPIs, androgen receptor pathway inhibitors; BPs, bisphosphonates; CDK4/6 inhibitors, cyclin-dependent

kinase 4/6 inhibitors; ERPAs, estrogen/progesterone receptor pathway agents; FAERS, FDA Adverse Event Reporting System; GnRH agonists, gonadotropin-releasing hormone agonists; ICIs, immune checkpoint inhibitors; ILD, interstitial lung disease; IQR, interquartile range; mAbs, monoclonal antibodies; MTIs, microtubule inhibitors; PARP inhibitors, poly(ADP-ribose) polymerase inhibitors; PBC, platinum-based chemotherapy; PI3K/AKT/mTOR inhibitors, PI3K/AKT/mTOR pathway inhibitors; SMD, standardized mean difference; TKIs, tyrosine kinase inhibitors; TTO, time to onset.

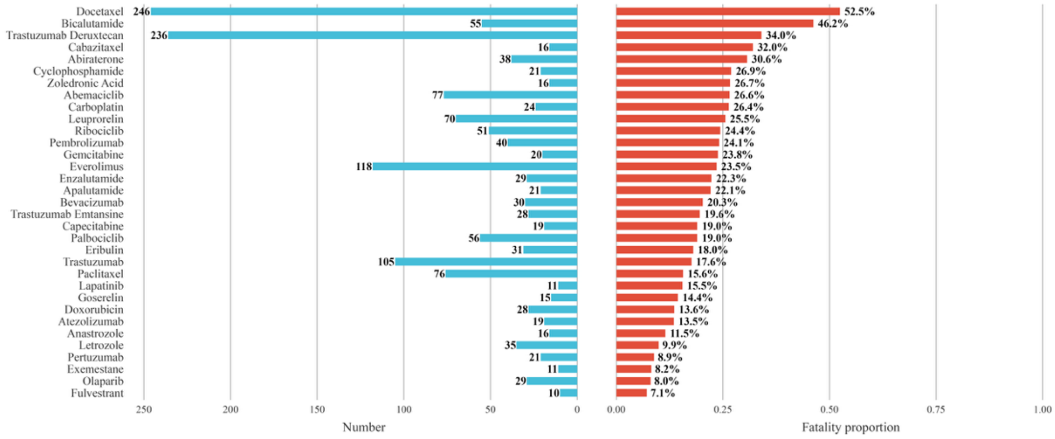

Figure S3: **Drug-specific counts and proportions of ILD reports with death recorded among therapies for sex hormone-sensitive solid tumors.** Notes: Drugs with ≥10 ILD reports with death recorded were summarized. The left panel shows the number of ILD reports with death recorded for each drug; the right panel shows the proportion of ILD reports with death recorded among all ILD reports for that drug. Across the 33 drugs included, there were 7,260 ILD reports, of which 1,618 had death recorded, corresponding to an overall death-recorded proportion of 22.3%. In FAERS, death indicates a reported serious outcome and does not necessarily establish that ILD was the confirmed cause of death. Drug-specific proportions should therefore be interpreted as reporting-based descriptive measures rather than incidence rates or comparative mortality risks. Abbreviations: FAERS, FDA Adverse Event Reporting System; ILD, interstitial lung disease.

In addition, we descriptively summarized ILD reports with death recorded in FAERS. The analysis included 33 drugs with ≥10 death-recorded ILD reports. Across these drugs, there were 7,260 ILD reports, of which 1,618 had death recorded, corresponding to a death-recorded proportion of 22.3%. When ordered descriptively by the proportion of ILD reports with death recorded, the largest observed proportions were for docetaxel, bicalutamide, and trastuzumab deruxtecan (T-DXd), followed by cabazitaxel and abiraterone (Figure S3).

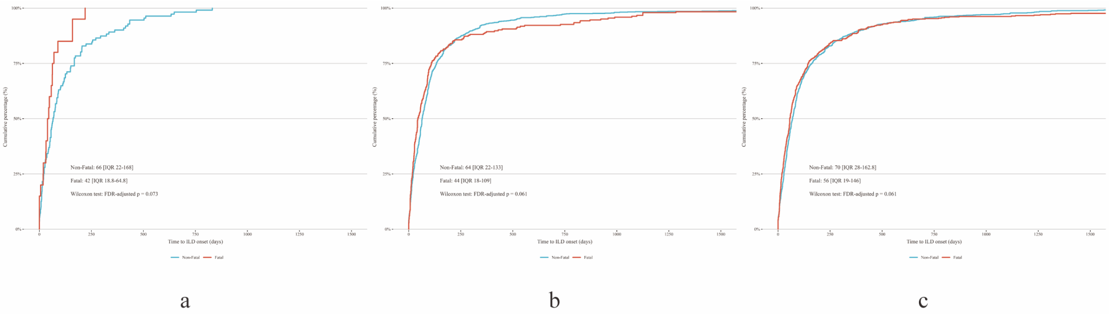

Figure S4: **ECDF curves for the time to onset of ILD following antineoplastic therapy for sex hormone-sensitive solid tumors: death-recorded reports vs non‑death-recorded reports by age subgroup.** Notes: Panels display ECDF curves comparing the distributions of ILD reports with and without recorded death outcomes within (a) the subgroup aged ≤40 years, (b) the subgroup aged >40 and ≤65 years, and (c) the subgroup aged >65 years. Group differences in reported TTO were evaluated using two-sided Wilcoxon rank-sum tests, and the panel-level *P* values shown for age subgroup comparisons are FDR-adjusted *P* values calculated using the Benjamini‑Hochberg method. Death-recorded reports were defined as reports with death recorded as the serious outcome in FAERS; this does not necessarily indicate that ILD was the confirmed cause of death. Age subgroup analyses were exploratory. Abbreviations: ECDF, empirical cumulative distribution function; FAERS, FDA Adverse Event Reporting System; FDR, false discovery rate; ILD, interstitial lung disease; TTO, time to onset.

Reported time-to-onset pattern and exploratory analysis of factors associated with reported onset timing

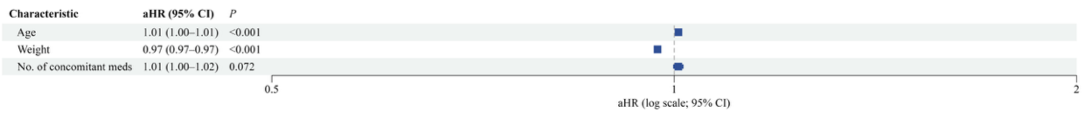

Figure S5: **Exploratory multivariable Cox analysis of reported time to onset of ILD after antineoplastic therapy for sex hormone-sensitive solid tumors.** Notes: The model was fitted among complete-case ILD reports with analyzable TTO and included age, body weight, and the number of concomitant medications. Estimates describe associations with reported onset timing and should not be interpreted as incidence, absolute risk, or causal risk factors for ILD. Abbreviations: aHR, adjusted hazard ratio; CI, confidence interval; ILD, interstitial lung disease; No., number; meds, medications; TTO, time to onset.

Building on the above analyses, we fitted an exploratory multivariable Cox proportional hazards model among ILD reports with analyzable TTO, including age, body weight, and the number of concomitant medications as covariates. In this complete-case analysis, older age was associated with earlier reported ILD onset (aHR, 1.01; $P < 0.001$), whereas higher body weight was associated with later reported onset (aHR, 0.97; $P < 0.001$). The number of concomitant medications showed a nominal association with earlier reported onset, but this association did not reach statistical significance (aHR, 1.01; $P = 0.072$) (Figure S5).

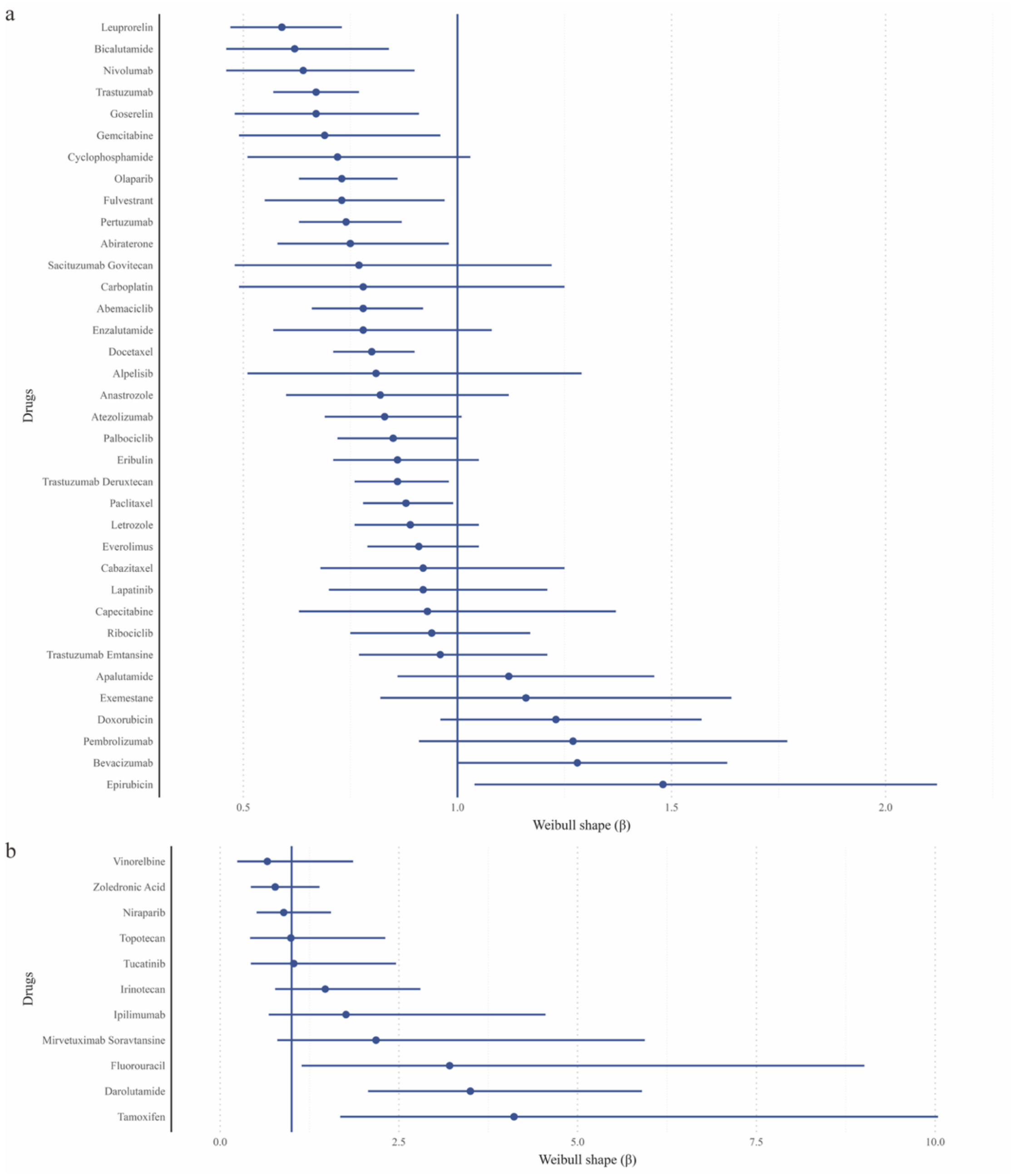

Figure S6: (a) **Weibull shape parameter β for reported time-to-onset patterns among drug–ILD pairs with ≥10 analyzable TTO reports;** (b) **Weibull shape parameter β for reported time-to-onset patterns among drug–ILD pairs with <10 analyzable TTO reports.** Notes: Weibull models were fitted for drug–ILD pairs with ≥3 nonmissing TTO values and at least two distinct TTO values. Points show β estimates with 95% CIs; the vertical line indicates β = 1. Reported onset patterns were classified by the 95% CI of β: early pattern if the upper bound was <1, random pattern if the CI included 1, and wear-out pattern if the lower bound was >1. Panel (b) is presented descriptively because of the small number of analyzable TTO reports. These classifications describe reported onset-time distributions among complete-case FAERS reports and should not be interpreted as true drug-specific hazard processes or causal failure mechanisms. Abbreviations: β, Weibull shape parameter; CI, confidence interval; FAERS, FDA Adverse Event Reporting System; ILD, interstitial lung disease; TTO, time to onset.

Finally, we performed a Weibull-based analysis to describe reported TTO patterns for drug–ILD pairs with analyzable onset-time data. Figure S6a presents drug–ILD pairs with ≥10 analyzable TTO reports, whereas Figure S6b presents drug–ILD pairs with <10 analyzable TTO reports and is provided descriptively. Among drug–ILD pairs with ≥10 analyzable TTO reports, 20 were classified as random pattern, 15 as early pattern, and one as wear-out pattern. Random patterns were observed for alpelisib, anastrozole, apalutamide, atezolizumab, bevacizumab, cabazitaxel, capecitabine, carboplatin, cyclophosphamide, doxorubicin, enzalutamide, eribulin, everolimus, exemestane, lapatinib, letrozole, pembrolizumab, ribociclib, sacituzumab govitecan, and trastuzumab emtansine. Early patterns were observed for abemaciclib, abiraterone, bicalutamide, docetaxel, fulvestrant, gemcitabine, goserelin, leuprorelin, nivolumab, olaparib, paclitaxel, palbociclib, pertuzumab, trastuzumab, and trastuzumab deruxtecan (T-DXd). Among drug–ILD pairs with ≥10 analyzable TTO reports, epirubicin was the only pair classified as having a wear-out pattern. Among drug–ILD pairs with <10 analyzable TTO reports, most were classified as random pattern, whereas fluorouracil, darolutamide, and tamoxifen were classified as wear-out pattern; these small-sample classifications were considered descriptive and hypothesis-generating.

Table S6: **Case-level summary of published interstitial lung disease reports involving the prespecified antineoplastic agents**

Case No.	Cancer type	Sex	Age (years)	PS regimen	Concomitant therapy	Concomitant SHA(s)	Outcome	Time to ILD onset (days)
1	Prostate cancer	Male	79	nilutamide	—	—	Not reported	14
2	Breast cancer	Female	62	cyclophosphamide + methotrexate + fluorouracil	—	—	ICU admission	14
3	Breast cancer	Female	71	trastuzumab	paclitaxel; doxorubicin; docetaxel; letrozole	letrozole	Hospitalization	131
4	Breast cancer	Female	58	gemcitabine	doxorubicin; paclitaxel	—	Hospitalization	140
5	Prostate cancer	Male	75	flutamide + leuprorelin	—	—	Death	8
6	Prostate cancer	Male	78	docetaxel	thalidomide	—	Hospitalization	56
7	Breast cancer	Female	NR	trastuzumab	epirubicin; cyclophosphamide; paclitaxel	—	Hospitalization	56

8	Breast cancer	Female	51	paclitaxel	doxorubicin; cyclophosphamide	—	Hospitalization	7
9	Breast cancer	Female	41	paclitaxel	doxorubicin; cyclophosphamide	—	Hospitalization	14
10	Breast cancer	Female	58	paclitaxel	—	—	Hospitalization	21
11	Ovarian cancer	Female	56	gemcitabine	—	—	Medical intervention	NR
12	Breast cancer	Female	35	docetaxel	doxorubicin; cyclophosphamide	—	Death	74
13	Ovarian cancer	Female	58	thalidomide	topotecan	—	Hospitalization	NR
14	Breast cancer	Male	41	cyclophosphamide	doxorubicin; zoledronic acid; tamoxifen	tamoxifen	Medical intervention	365
15	Breast and ovarian cancer	Female	51	cyclophosphamide	tamoxifen; fluorouracil; methotrexate; carboplatin; etoposide	tamoxifen	Hospitalization	NR
16	Breast cancer	Female	59	trastuzumab	paclitaxel	—	Hospitalization	112
17	Ovarian cancer	Female	18	bleomycin	cisplatin; vinblastine	—	Medical intervention	1230

18	Ovarian cancer	Female	26	bleomycin	cisplatin; vinblastine	—	Medical intervention	NR
19	Breast cancer	Female	67	medroxyprogesterone	—	—	Death	42
20	Prostate cancer	Male	55	apalutamide	triptorelin	triptorelin	Medical intervention	30
21	Prostate cancer	Male	73	apalutamide	leuprorelin	leuprorelin	Medical intervention	180
22	Breast cancer	Female	44	paclitaxel	doxorubicin	—	Hospitalization	7
23	Prostate cancer	Male	69	nilutamide	—	—	Hospitalization	60
24	Breast cancer	Female	47	everolimus	exemestane	exemestane	Hospitalization	NR
25	Breast cancer	Female	60	docetaxel	—	—	Death	NR
26	Breast cancer	Female	58	cyclophosphamide	tamoxifen	tamoxifen	Death	730
27	Breast cancer	Female	48	docetaxel	doxorubicin; cyclophosphamide	—	Hospitalization	21
28	Breast cancer	Female	77	abemaciclib	—	—	Hospitalization	30
29	Breast cancer	Female	53	bevacizumab	doxorubicin	—	Hospitalization	42
30	Ovarian cancer	Female	48	doxorubicin	—	—	Medical intervention	23

31	Ovarian cancer	Female	72	olaparib	bevacizumab	—	Hospitalization	120
32	Ovarian cancer	Female	51	olaparib	—	—	Medical intervention	270
33	Ovarian cancer	Female	78	olaparib	—	—	Hospitalization	14
34	Breast cancer	Female	65	abemaciclib	fulvestrant	fulvestrant	Hospitalization	105
35	Breast cancer	Female	49	trastuzumab	capecitabine	—	Medical intervention	180
36	Ovarian cancer	Female	58	ipilimumab	—	—	Medical intervention	343
37	Breast cancer	Female	55	pembrolizumab	—	—	Hospitalization	21
38	Breast cancer	Female	65	everolimus	—	—	Hospitalization	120
39	Breast cancer	Female	71	docetaxel	—	—	Hospitalization	NR
40	Breast cancer	Female	46	docetaxel + cyclophosphamide	doxorubicin	—	Hospitalization	63
41	Breast cancer	Female	59	gemcitabine + paclitaxel	doxorubicin; cyclophosphamide	—	Hospitalization	NR
42	Breast cancer	Female	64	docetaxel	—	—	Death	45

43	Breast cancer	Female	48	docetaxel	—	—	Hospitalization	19
44	Breast cancer	Female	68	everolimus	exemestane	exemestane	Hospitalization	84
45	Ovarian cancer	Female	83	gemcitabine	—	—	Hospitalization	56
46	Breast cancer	Female	42	capecitabine	—	—	Hospitalization	NR
47	Prostate cancer	Male	71	apalutamide	—	—	Hospitalization	90
48	Prostate cancer	Male	84	apalutamide	—	—	Hospitalization	90
49	Breast cancer	Female	46	pembrolizumab	gemcitabine; carboplatin	—	Hospitalization	15
50	Breast cancer	Female	64	abemaciclib	letrozole	letrozole	Medical intervention	365
51	Breast cancer	Female	84	pembrolizumab	—	—	Medical intervention	104
52	Breast cancer	Female	63	docetaxel	trastuzumab	—	Death	NR
53	Prostate cancer	Male	73	docetaxel	abiraterone	abiraterone	Medical intervention	NR
54	Breast cancer	Female	70	trastuzumab	—	—	Death	NR

55	Prostate cancer	Male	72	docetaxel	—	—	Death	NR
56	Breast cancer	Female	62	paclitaxel	—	—	Medical intervention	NR
57	Breast cancer	Female	62	sacituzumab govitecan	—	—	Medical intervention	NR
58	Prostate cancer	Male	75	bicalutamide + leuprorelin	—	—	Death	14
59	Breast cancer	Female	70	doxorubicin	letrozole; fulvestrant	letrozole; fulvestrant	Death	450
60	Prostate cancer	Male	78	bicalutamide	—	—	Hospitalization	240
61	Breast cancer	Female	57	paclitaxel	trastuzumab	—	Medical intervention	210
62	Breast cancer	Female	48	cyclophosphamide + docetaxel	goserelin; doxorubicin	goserelin	Hospitalization	NR
63	Breast cancer	Female	41	cyclophosphamide + docetaxel	—	—	Hospitalization	10
64	Prostate cancer	Male	72	docetaxel	estramustine	—	Death	112
65	Prostate	Male	76	docetaxel	estramustine;	—	Death	NR

	cancer				doxorubicin			
66	Breast cancer	Female	72	eribulin	anastrozole; tamoxifen; medroxyprogesterone; fulvestrant; capecitabine; doxorubicin; cyclophosphamide; paclitaxel	anastrozole; tamoxifen; medroxyprogesterone; fulvestrant	Hospitalization	5
67	Breast cancer	Female	73	eribulin	exemestane; anastrozole; tamoxifen; toremifene; letrozole; medroxyprogesterone; fulvestrant; capecitabine; paclitaxel	exemestane; anastrozole; tamoxifen; toremifene; letrozole; medroxyprogesterone; fulvestrant	Hospitalization	168
68	Breast cancer	Female	72	eribulin	letrozole; paclitaxel; tamoxifen; fulvestrant; doxorubicin; cyclophosphamide; capecitabine	letrozole; tamoxifen; fulvestrant	Hospitalization	7
69	Breast cancer	Female	66	paclitaxel	—	—	Hospitalization	10

70	Prostate cancer	Male	88	fulvestrant	—	—	Hospitalization	21
71	Breast cancer	Female	40	abemaciclib	fulvestrant	fulvestrant	Hospitalization	300
72	Breast cancer	Female	89	palbociclib	fulvestrant	fulvestrant	Death	90
73	Breast cancer	Female	60	epirubicin + cyclophosphamide	—	—	Hospitalization	NR
74	Ovarian cancer	Female	62	paclitaxel	capecitabine	—	Death	180
75	Breast cancer	Female	61	docetaxel	doxorubicin; cyclophosphamide	—	Hospitalization	60
76	Ovarian cancer	Female	58	gemcitabine	—	—	Death	NR
77	Breast cancer	Female	56	trastuzumab	docetaxel	—	Hospitalization	150
78	Breast cancer	Female	49	trastuzumab	docetaxel	—	Hospitalization	42
79	Breast cancer	Female	63	ribociclib	letrozole; zoledronic acid	letrozole	Hospitalization	120
80	Prostate cancer	Male	73	docetaxel	—	—	Hospitalization	60
81	Breast cancer	Female	52	cyclophosphamide	epirubicin; fluorouracil	—	Hospitalization	6

82	Prostate cancer	Male	72	bicalutamide + leuprorelin	—	—	Hospitalization	60
83	Ovarian cancer	Female	73	niraparib	—	—	Hospitalization	47
84	Breast cancer	Female	58	docetaxel	epirubicin	—	Death	NR
85	Breast cancer	Female	54	trastuzumab deruxtecan	—	—	Hospitalization	8
86	Breast cancer	Female	47	carmustine	cyclophosphamide; cisplatin	—	Hospitalization	42
87	Breast cancer	Female	50	atezolizumab + bevacizumab	paclitaxel	—	Hospitalization	NR
88	Breast cancer	Female	59	cyclophosphamide	doxorubicin	—	Hospitalization	NR
89	Ovarian cancer	Female	40	cyclophosphamide	—	—	Death	480
90	Breast cancer	Female	47	trastuzumab	tamoxifen	tamoxifen	Hospitalization	NR
91	Breast cancer	Female	72	trastuzumab	tamoxifen	tamoxifen	Hospitalization	90
92	Prostate cancer	Male	94	leuprorelin	bicalutamide	bicalutamide	Hospitalization	NR
93	Prostate cancer	Male	68	nilutamide + leuprorelin	—	—	Hospitalization	60

94	Breast cancer	Female	61	paclitaxel	cyclophosphamide; doxorubicin; fluorouracil	—	Hospitalization	7
95	Breast cancer	Female	79	lapatinib	letrozole	letrozole	Hospitalization	12
96	Breast cancer	Female	41	paclitaxel	trastuzumab	—	Hospitalization	30
97	Prostate cancer	Male	66	cabazitaxel	enzalutamide; docetaxel	enzalutamide	Hospitalization	21
98	Breast cancer	Female	72	anastrozole	—	—	Death	2190

Notes: Cases were summarized using standardized drug names. PS combination regimens were recorded as reported in the source publications. Time-to-onset values were harmonized and reported in days. Abbreviations: ILD, interstitial lung disease; PS, primary suspect; SHA, sex hormone-pathway agent.

Supplementary notes on exploratory analyses

In this FAERS cohort, 22.3% of ILD reports included in the drug-level outcome summary had death recorded. This proportion should be interpreted cautiously because severe outcomes may be more likely to be reported, and FAERS does not establish whether ILD was the confirmed cause of death. Nevertheless, the substantial proportion of death-recorded reports underscores the clinical seriousness of ILD reports associated with antineoplastic therapy and supports the need for careful pharmacovigilance and clinical awareness.

The Weibull-based analysis suggested heterogeneous reported onset-time patterns across drug–ILD pairs. These findings should be interpreted as descriptive reporting patterns among complete-case FAERS reports, rather than as estimates of true drug-specific hazard processes. Among drug–ILD pairs with ≥10 analyzable TTO reports, 15 were classified as early pattern, suggesting that reported ILD onset tended to occur relatively early after treatment initiation in these reports. Trastuzumab deruxtecan was classified as early pattern (β = 0.86; 95% CI, 0.76–0.98), broadly consistent with clinical trial observations that ILD/pneumonitis with trastuzumab deruxtecan often occurs within the first several months of treatment.[2] Abemaciclib was also classified as early pattern (β = 0.78; 95% CI, 0.66–0.92), which is compatible with clinical observations of pneumonitis during the first year of therapy.[3] In contrast, several agents showed random patterns, including ribociclib (β = 0.94; 95% CI, 0.75–1.17) and pembrolizumab (β = 1.27; 95% CI, 0.91–1.77), indicating that reported onset was not concentrated within a clearly defined early or late period in the available FAERS TTO data. Epirubicin was classified as wear-out pattern among drug–ILD pairs with ≥10 analyzable TTO reports (β = 1.50; 95% CI, 1.04–2.12). Wear-out classifications in smaller samples, such as tamoxifen and darolutamide, should be interpreted cautiously because of limited report counts and greater statistical instability. Overall, these findings may help identify reporting patterns that warrant clinical awareness, but they should not be used to define fixed monitoring intervals or to infer causal time-dependent risk.

References

1. US Food and Drug Administration. Considerations for the Inclusion of Adolescent Patients in Adult Oncology Clinical Trials: Guidance for Industry. Silver Spring, MD: US Food and Drug Administration; 2019. Accessed May 12, 2026. https://www.fda.gov/media/113499/download.
2. Modi S, Jacot W, Iwata H, Park YH, Vidal Losada M, Li W, et al. Trastuzumab deruxtecan in HER2-low metastatic breast cancer: long-term survival analysis of the randomized, phase 3 DESTINY-Breast04 trial. Nat Med. 2025;31(12):4205-4213. doi:10.1038/s41591-025-03981-4
3. Hashimoto T, Nakamura H, Sakoda Y, Tsuchiya K, Fujii M, Taki M, et al. The Incidence of Abemaciclib-induced Interstitial Lung Disease: A Single-center Retrospective Study in Japan. Kobe Journal of Medical Sciences. Preprint posted online 2025:2. doi:10.24546/0100497175
