# Supplementary material for: Antineoplastic agent-associated interstitial lung disease in breast, ovarian, and prostate cancers: a pharmacovigilance study using the FDA adverse event reporting system
Source: Front Immunol. 2026 Jun 10;17:1840323. doi: 10.3389/fimmu.2026.1840323 (PMC13290764; doi:10.3389/fimmu.2026.1840323)
Supplement: Supplementary file 2 [file DataSheet2.docx]

PubMed search strategies

PubMed search date: March 15, 2026

Coverage: From database inception to March 15, 2026

**Search strategy 1**

ILD × broad antineoplastic treatment concepts × case reports/case series × humans

("Lung Diseases, Interstitial"[Mesh] OR "Pneumonitis"[Mesh] OR "Pulmonary Fibrosis"[Mesh] OR

"Pneumonia, Organizing"[Mesh] OR "Hypersensitivity Pneumonitis"[Mesh]

OR "interstitial lung disease"[tiab] OR ILD[tiab] OR pneumonitis[tiab] OR

"immune-related pneumonitis"[tiab] OR "immune mediated pneumonitis"[tiab] OR

"immune-mediated pneumonitis"[tiab]

OR "drug-induced interstitial lung disease"[tiab] OR "drug-induced pneumonitis"[tiab] OR

"interstitial pneumonitis"[tiab] OR "interstitial pneumonia"[tiab] OR

"organizing pneumonia"[tiab] OR "pulmonary fibrosis"[tiab]

OR "diffuse alveolar damage"[tiab] OR "acute interstitial pneumonitis"[tiab] OR

"alveolar lung disease"[tiab] OR "alveolar proteinosis"[tiab] OR alveolitis[tiab] OR

"alveolitis necrotising"[tiab]

OR "autoimmune lung disease"[tiab] OR bronchiolitis[tiab] OR

"bronchiolitis obliterans syndrome"[tiab] OR "chronic graft versus host disease in lung"[tiab]

OR "combined pulmonary fibrosis and emphysema"[tiab] OR

"confirmed e-cigarette or vaping product use associated lung injury"[tiab] OR

"eosinophilia myalgia syndrome"[tiab]

OR "eosinophilic granulomatosis with polyangiitis"[tiab] OR

"eosinophilic pneumonia"[tiab] OR "eosinophilic pneumonia acute"[tiab] OR

"eosinophilic pneumonia chronic"[tiab]

OR "hypersensitivity pneumonitis"[tiab] OR "idiopathic interstitial pneumonia"[tiab] OR

"idiopathic pneumonia syndrome"[tiab] OR "idiopathic pulmonary fibrosis"[tiab]

OR "immune-mediated lung disease"[tiab] OR "interstitial lung abnormality"[tiab] OR

"low lung compliance"[tiab] OR "lung infiltration"[tiab] OR "lung opacity"[tiab]

OR "necrotising bronchiolitis"[tiab] OR "obliterative bronchiolitis"[tiab] OR

"pleuro parenchymal fibroelastosis"[tiab]

OR "probable e-cigarette or vaping product use associated lung injury"[tiab] OR

"progressive massive fibrosis"[tiab] OR "pulmonary necrosis"[tiab]

OR "pulmonary radiation injury"[tiab] OR "pulmonary toxicity"[tiab] OR

"pulmonary vasculitis"[tiab] OR "radiation alveolitis"[tiab] OR "radiation bronchitis"[tiab]

OR "radiation fibrosis - lung"[tiab] OR "radiation pneumonitis"[tiab] OR

"rheumatoid arthritis-associated interstitial lung disease"[tiab]

OR "small airways disease"[tiab] OR "transfusion-related acute lung injury"[tiab])

AND

("Antineoplastic Agents"[Mesh]

OR antineoplastic*[tiab] OR chemotherap*[tiab] OR "cancer therap*"[tiab] OR

anticancer[tiab] OR "anti-cancer"[tiab]

OR immunotherap*[tiab] OR "immune checkpoint"[tiab] OR "checkpoint inhibitor*"[tiab] OR

"immune checkpoint inhibitor*"[tiab]

OR ICI[tiab] OR PD-1[tiab] OR PD-L1[tiab] OR CTLA-4[tiab]

OR "antibody-drug conjugate*"[tiab] OR "antibody drug conjugate*"[tiab] OR

ADC[tiab]

OR "monoclonal antibod*"[tiab] OR "targeted therap*"[tiab]

OR "endocrine therap*"[tiab] OR "hormone therap*"[tiab] OR

"hormonal therap*"[tiab] OR "androgen deprivation"[tiab])

AND

("Case Reports"[pt] OR case report*[tiab] OR case series[tiab] OR case stud*[tiab])

AND

"Humans"[Mesh]

**Search strategy 2**

ILD × prespecified antineoplastic agents × case reports/case series × humans

("Lung Diseases, Interstitial"[Mesh] OR "Pneumonitis"[Mesh] OR "Pulmonary Fibrosis"[Mesh] OR

"Pneumonia, Organizing"[Mesh] OR "Hypersensitivity Pneumonitis"[Mesh]

OR "interstitial lung disease"[tiab] OR ILD[tiab] OR pneumonitis[tiab] OR

"immune-related pneumonitis"[tiab] OR "immune mediated pneumonitis"[tiab] OR

"immune-mediated pneumonitis"[tiab]

OR "drug-induced interstitial lung disease"[tiab] OR "drug-induced pneumonitis"[tiab] OR

"interstitial pneumonitis"[tiab] OR "interstitial pneumonia"[tiab] OR

"organizing pneumonia"[tiab] OR "pulmonary fibrosis"[tiab]

OR "diffuse alveolar damage"[tiab] OR "acute interstitial pneumonitis"[tiab] OR

"alveolar lung disease"[tiab] OR "alveolar proteinosis"[tiab] OR alveolitis[tiab] OR

"alveolitis necrotising"[tiab]

OR "autoimmune lung disease"[tiab] OR bronchiolitis[tiab] OR

"bronchiolitis obliterans syndrome"[tiab] OR "chronic graft versus host disease in lung"[tiab]

OR "combined pulmonary fibrosis and emphysema"[tiab] OR

"confirmed e-cigarette or vaping product use associated lung injury"[tiab] OR

"eosinophilia myalgia syndrome"[tiab]

OR "eosinophilic granulomatosis with polyangiitis"[tiab] OR

"eosinophilic pneumonia"[tiab] OR "eosinophilic pneumonia acute"[tiab] OR

"eosinophilic pneumonia chronic"[tiab]

OR "hypersensitivity pneumonitis"[tiab] OR "idiopathic interstitial pneumonia"[tiab] OR

"idiopathic pneumonia syndrome"[tiab] OR "idiopathic pulmonary fibrosis"[tiab]

OR "immune-mediated lung disease"[tiab] OR "interstitial lung abnormality"[tiab] OR

"low lung compliance"[tiab] OR "lung infiltration"[tiab] OR "lung opacity"[tiab]

OR "necrotising bronchiolitis"[tiab] OR "obliterative bronchiolitis"[tiab] OR

"pleuro parenchymal fibroelastosis"[tiab]

OR "probable e-cigarette or vaping product use associated lung injury"[tiab] OR

"progressive massive fibrosis"[tiab] OR "pulmonary necrosis"[tiab]

OR "pulmonary radiation injury"[tiab] OR "pulmonary toxicity"[tiab] OR

"pulmonary vasculitis"[tiab] OR "radiation alveolitis"[tiab] OR "radiation bronchitis"[tiab]

OR "radiation fibrosis - lung"[tiab] OR "radiation pneumonitis"[tiab] OR

"rheumatoid arthritis-associated interstitial lung disease"[tiab]

OR "small airways disease"[tiab] OR "transfusion-related acute lung injury"[tiab])

AND

("trastuzumab deruxtecan"[tiab] OR trastuzumab[tiab] OR everolimus[tiab] OR

paclitaxel[tiab] OR docetaxel[tiab] OR olaparib[tiab] OR letrozole[tiab]

OR palbociclib[tiab] OR abemaciclib[tiab] OR leuprorelin[tiab] OR pertuzumab[tiab]

OR ribociclib[tiab] OR doxorubicin[tiab] OR eribulin[tiab] OR pembrolizumab[tiab]

OR "trastuzumab emtansine"[tiab] OR bevacizumab[tiab] OR atezolizumab[tiab]

OR anastrozole[tiab] OR fulvestrant[tiab] OR exemestane[tiab] OR enzalutamide[tiab]

OR abiraterone[tiab] OR bicalutamide[tiab] OR niraparib[tiab] OR goserelin[tiab]

OR capecitabine[tiab] OR epirubicin[tiab] OR apalutamide[tiab] OR carboplatin[tiab]

OR gemcitabine[tiab] OR cyclophosphamide[tiab] OR lapatinib[tiab]

OR "zoledronic acid"[tiab] OR cabazitaxel[tiab] OR "sacituzumab govitecan"[tiab]

OR darolutamide[tiab] OR fluorouracil[tiab] OR alpelisib[tiab] OR tamoxifen[tiab]

OR nivolumab[tiab] OR denosumab[tiab] OR vinorelbine[tiab] OR tucatinib[tiab]

OR "mirvetuximab soravtansine"[tiab] OR ipilimumab[tiab] OR topotecan[tiab]

OR flutamide[tiab] OR nilutamide[tiab] OR cisplatin[tiab]

OR "pertuzumab trastuzumab vorhyaluronidase alfa"[tiab] OR estramustine[tiab]

OR toremifene[tiab] OR capivasertib[tiab] OR etoposide[tiab] OR irinotecan[tiab]

OR rucaparib[tiab] OR lenalidomide[tiab] OR triptorelin[tiab] OR talazoparib[tiab]

OR durvalumab[tiab] OR toripalimab[tiab] OR neratinib[tiab]

OR medroxyprogesterone[tiab] OR "pertuzumab trastuzumab"[tiab])

AND

("Case Reports"[pt] OR case report*[tiab] OR case series[tiab] OR case stud*[tiab])

AND

"Humans"[Mesh]
