# Supplementary material for: Antineoplastic agent-associated interstitial lung disease in breast, ovarian, and prostate cancers: a pharmacovigilance study using the FDA adverse event reporting system
Source: Front Immunol. 2026 Jun 10;17:1840323. doi: 10.3389/fimmu.2026.1840323 (PMC13290764; doi:10.3389/fimmu.2026.1840323)
Supplement: Supplementary file 3 [file DataSheet3.docx]

Source publications included in the structured literature review

1. Abdul Hamid MF, Che Rahim MJ, Arif N, Mohamad Jailaini MF. Sequential radiation-induced and immune checkpoint inhibitor-induced pneumonitis in a patient with breast carcinoma. BMJ Case Rep. 2026;19(1):e269851. doi:10.1136/bcr-2025-269851

2. Akoun GM, Liote HA, Liote F, Gauthier-Rahman S, Kuntz D. Provocation test coupled with bronchoalveolar lavage in diagnosis of drug (nilutamide)-induced hypersensitivity pneumonitis. Chest. 1990;97(2):495-498. doi:10.1378/chest.97.2.495

3. Al-Hameed FM. Saddle pulmonary embolus and bronchiolitis obliterans with organizing pneumonia develop simultaneously after first cyclophosphamide, methotrexate, 5FU chemotherapy for breast cancer. Saudi Med J. 2015;36(6):747-750. doi:10.15537/smj.2015.6.11305

4. Alkan A. Interstitial pneumonitis associated with trastuzumab emtansine. J Oncol Pharm Pract. 2019;25(7):1798-1800. doi:10.1177/1078155218813716

5. Antolín S, Calvo L, Dopico D, Quindós M, Reboredo M, Carral A. Locally advanced breast cancer: pulmonary toxicity secondary to gemcitabine. Clin Transl Oncol. 2010;12(6):450-452. doi:10.1007/s12094-010-0533-5

6. Azuma T, Kurimoto S, Mikami K, Oshi M. Interstitial pneumonitis related to leuprorelin acetate and flutamide. J Urol. 1999;161(1):221.

7. Behrens RJ, Gulley JL, Dahut WL. Pulmonary toxicity during prostate cancer treatment with docetaxel and thalidomide. Am J Ther. 2003;10(3):228-232. doi:10.1097/00045391-200305000-00011

8. Bettini AC, Tondini C, Poletti P, Caremoli ER, Guerra U, Labianca R. A case of interstitial pneumonitis associated with Guillain-Barré syndrome during administration of adjuvant trastuzumab. Tumori. 2008;94(5):737-741. doi:10.1177/030089160809400516

9. Bielopolski D, Evron E, Moreh-Rahav O, Landes M, Stemmer SM, Salamon F. Paclitaxel-induced pneumonitis in patients with breast cancer: case series and review of the literature. J Chemother. 2017;29(2):113-117. doi:10.1179/1973947815Y.0000000029

10. Boiselle PM, Morrin MM, Huberman MS. Gemcitabine pulmonary toxicity: CT features. J Comput Assist Tomogr. 2000;24(6):977-980. doi:10.1097/00004728-200011000-00027

11. Brahmi SA, Youssef S, Ziani FZ, Afqir S. [Fatal acute interstitial lung disease associated with docetaxel administration: about a case and review of the literature]. Pan Afr Med J. 2016;24:119. doi:10.11604/pamj.2016.24.119.8902

12. Buttin BM, Moore MJ. Thalidomide-induced reversible interstitial pneumonitis in a patient with recurrent ovarian cancer. Gynecol Oncol. 2008;111(3):546-548. doi:10.1016/j.ygyno.2007.11.005

13. Buyse V, Brusselle G, Cocquyt V, Van Belle S. A 41-year-old man with breast cancer and dyspnoea: an exceptional complication in an uncommon disease. Acta Clin Belg. 2010;65(5):341-344. doi:10.1179/acb.2010.073

14. Callaway MP, Tyrrell CJ, Williams MP, Marshall AJ. Chemotherapy induced myocardial fibrosis. Clin Oncol (R Coll Radiol). 1994;6(1):55-56. doi:10.1016/s0936-6555(05)80369-3

15. Chang CH, Jung CJ, Huang YM, Chiao L, Chang YL, Hsieh SC, et al. The first reported case of trastuzumab induced interstitial lung disease associated with anti-neutrophil cytoplasmic antibody vasculitis - A case report and a prospective cohort study on the usefulness of neutrophil derived biomarkers in monitoring vasculitis disease activity during follow-up. Breast. 2022;61:35-42. doi:10.1016/j.breast.2021.11.016

16. Davis TE, Loprinzi CL, Buchler DA. Combination chemotherapy with cisplatin, vinblastine, and bleomycin for endodermal sinus tumor of the ovary. Gynecol Oncol. 1984;19(1):46-52. doi:10.1016/0090-8258(84)90156-2

17. De Greve J, Warson F, Deleu D, Storme G. Fatal pulmonary toxicity by the association of radiotherapy and medroxyprogesterone acetate. Cancer. 1985;56(10):2434-2436. doi:10.1002/1097-0142(19851115)56:10%3C2434::aid-cncr2820561018%3E3.0.co;2-p

18. Del Castillo-Acuña R, Serradilla A, López-Campos F, Couñago F. Interstitial Lung Disease Caused by Apalutamide for Metastatic Castration-Sensitive Prostate Cancer. Arch Bronconeumol. 2024;60(8):537-538. doi:10.1016/j.arbres.2024.04.009

19. Goldberg HL, Vannice SB. Pneumonitis related to treatment with paclitaxel. J Clin Oncol. 1995;13(2):534-535. doi:10.1200/JCO.1995.13.2.534

20. Gomez JL, Dupont A, Cusan L, Tremblay M, Tremblay M, Labrie F. Simultaneous liver and lung toxicity related to the nonsteroidal antiandrogen nilutamide (Anandron): a case report. Am J Med. 1992;92(5):563-566. doi:10.1016/0002-9343(92)90756-2

21. Gulde A, Hasanov E, Krug K, Krucke GW. Everolimus-induced pneumonitis: A diagnostic challenge. Breast J. 2020;26(2):287-288. doi:10.1111/tbj.13554

22. Gurram MK, Pulivarthi S, McGary CT. Fatal hypersensitivity pneumonitis associated with docetaxel. Tumori. 2013;99(3):e100-103. doi:10.1177/030089161309900325

23. Hamada K, Nagai S, Kitaichi M, Jin G, Shigematsu M, Nagao T, et al. Cyclophosphamide-induced late-onset lung disease. Intern Med. 2003;42(1):82-87. doi:10.2169/internalmedicine.42.82

24. Hirata A, Terasawa R, Takashima Y, Morita S, Kimura K, Iwamoto M, et al. [A Case of Severe Drug-Induced Lung Injury during Preoperative Chemotherapy for Breast Cancer in the Early Stages of the COVID-19 Epidemic]. Gan To Kagaku Ryoho. 2023;50(12):1343-1345.

25. Hirohashi Y, Kawashima Y, Shimonishi T, Koga Y, Miyake S, Hotta C, et al. [A Case of Recurrent Breast Cancer with Drug-Induced Interstitial Pneumonia Triggered by the Switch from an Original to a Generic Aromatase Inhibitor]. Gan To Kagaku Ryoho. 2020;47(12):1707-1709.

26. Hunt A, Haque W, Pino R, Farach A, Butler EB, Teh BS. Radiation Pneumonitis, Really? A Case of Pulmonary Toxicity from CDK4/6 Inhibitor. Anticancer Res. 2023;43(8):3539-3542. doi:10.21873/anticanres.16531

27. Huober J, Schoch O, Templeton A, Spirig C, Thürlimann B. Interstitial pneumonitis after treatment with bevacizumab and pegylated liposomal doxorubicin in a patient with metastatic breast cancer. Chemotherapy. 2010;56(1):69-70. doi:10.1159/000282286

28. Inaba K, Arimoto T, Hoya M, Kawana K, Nakagawa S, Kozuma S, et al. Interstitial pneumonitis induced by pegylated liposomal doxorubicin in a patient with recurrent ovarian cancer. Med Oncol. 2012;29(2):1255-1257. doi:10.1007/s12032-011-9893-0

29. Ishimoto H, Sakamoto N, Kido T, Ozasa M, Tsutsui S, Mori M, et al. Drug-induced interstitial lung disease caused by olaparib: three case reports and review of the Japanese Adverse Drug Event Report database and literature. BMC Pulm Med. 2023;23(1):289. doi:10.1186/s12890-023-02569-3

30. Isiklar A, Basaran G, Sepin B, Gumusay O, Kocagoz AS, Cuhadaroglu C. Alpelisib induced interstitial lung disease in a patient with advanced breast cancer. J Oncol Pharm Pract. 2023;29(2):484-488. doi:10.1177/10781552221107532

31. Jin F, Wang ST. Chronic eosinophilic pneumonia after trastuzumab and radiation therapy for breast cancer: A case report. Medicine (Baltimore). 2019;98(1):e14017. doi:10.1097/MD.0000000000014017

32. Johnson C, Jazaeri AA. Diagnosis and Management of Immune Checkpoint Inhibitor-related Toxicities in Ovarian Cancer: A Series of Case Vignettes. Clin Ther. 2018;40(3):389-394. doi:10.1016/j.clinthera.2018.02.011

33. Jun J, Lee SR, Lee JY, Choi MJ, Noh JY, Cheong HJ, et al. Pneumonitis and concomitant bacterial pneumonia in patients receiving pembrolizumab treatment: Three case reports and literature review. Medicine (Baltimore). 2019;98(25):e16158. doi:10.1097/MD.0000000000016158

34. Junpaparp P, Sharma B, Samiappan A, Rhee JH, Young KR. Everolimus-induced severe pulmonary toxicity with diffuse alveolar hemorrhage. Ann Am Thorac Soc. 2013;10(6):727-729. doi:10.1513/AnnalsATS.201309-332LE

35. Karacan O, Eyüboglu FO, Akçay S, Ozyilkan O. Acute interstitial pneumopathy associated with docetaxel hypersensitivity. Onkologie. 2004;27(6):563-565. doi:10.1159/000081339

36. Kim S, Tannock I, Sridhar S, Seki J, Bordeleau L. Chemotherapy-induced infiltrative pneumonitis cases in breast cancer patients. J Oncol Pharm Pract. 2012;18(2):311-315. doi:10.1177/1078155211429384

37. Kinoshita H, Teraoka H, Hasegawa T, Nakamoto K, Kashiwagi S, Hirakawa K, et al. [A Case of Metastatic Breast Cancer That Recovered from Diffuse Alveolar Damage Associated with Everolims]. Gan To Kagaku Ryoho. 2020;47(13):2370-2372.

38. Ko E, Lee S, Goodman A. Gemcitabine pulmonary toxicity in ovarian cancer. Oncologist. 2008;13(7):807-811. doi:10.1634/theoncologist.2008-0049

39. Kobayashi N, Tono Y, Tsunoda A, Oka H, Saito K, Yamashita Y, et al. [Interstitial Lung Disease after OK-432 Pleurodesis for Malignant Pleural Effusion in Breast Cancer-A Case Report]. Gan To Kagaku Ryoho. 2023;50(2):183-186.

40. Kobe H, Tachikawa R, Masuno Y, Matsunashi A, Murata S, Hagimoto H, et al. Apalutamide-induced severe interstitial lung disease: A report of two cases from Japan. Respir Investig. 2021;59(5):700-705. doi:10.1016/j.resinv.2021.05.006

41. Koi Y, Tajiri W, Kawasaki J, Akiyoshi S, Ijichi H, Nakamura Y, et al. A dramatic response to an immune checkpoint inhibitor plus chemotherapy in a patient with metastatic metaplastic carcinoma of the breast: A case report. Thorac Cancer. 2024;15(28):2073-2076. doi:10.1111/1759-7714.15433

42. Komoda A, Kashiwagi S, Kawano Y, Ishihara S, Goto W, Asano Y, et al. [A Case of Liver Abscess during Treatment for Abemaciclib-Induced Interstitial Lung Disease]. Gan To Kagaku Ryoho. 2022;49(1):100-102.

43. Köylü B, Kıkılı Cİ, Dikensoy Ö, Selçukbiricik F. Late-onset recurrent immune checkpoint inhibitor-related pneumonitis after cessation of pembrolizumab: a case report. Immunotherapy. 2025;17(5):317-320. doi:10.1080/1750743X.2025.2488609

44. Kuip E, Muller E. Fatal pneumonitis after treatment with docetaxel and trastuzumab. Neth J Med. 2009;67(6):237-239.

45. Kumar S, Singh H, Das CK, Kumar R, Mittal BR. Docetaxel-Induced Interstitial Pneumonitis Detected on 68Ga-PSMA PET/CT. Clin Nucl Med. 2021;46(5):e268-e269. doi:10.1097/RLU.0000000000003445

46. LaMorte D, Desmond D, Ellis J, Lipkowitz S. Acute eosinophilic pneumonia: a fatal reaction to ado-trastuzumab. BMJ Case Rep. 2021;14(9). doi:10.1136/bcr-2021-243881

47. Leimgruber K, Negro R, Baier S, Moser B, Resch G, Sansone S, et al. Fatal interstitial pneumonitis associated with docetaxel administration in a patient with hormone-refractory prostate cancer. Tumori. 2006;92(6):542-544. doi:10.1177/030089160609200614

48. Li A, Mohammadi F, Crocker H. Bilateral temporomandibular joint dislocations post-bronchoscopy in a case of paclitaxel-induced pneumonitis. BMJ Case Rep. 2021;14(2). doi:10.1136/bcr-2020-240146

49. Lin MC, Lin CW. Interstitial pneumonitis related to sacituzumab govitecan in a patient with metastatic triple-negative breast cancer: a case report. J Med Case Rep. 2026;20(1):103. doi:10.1186/s13256-025-05820-z

50. Maeda K, Osafune T, Masuda Y, Takeda T, Kageyama S, Narita M, et al. [DRUG-INDUCED INTERSTITIAL LUNG DISEASE DURING COMBINED ANDROGEN BLOCKADE WITH BICALUTAMIDE AND LEUPRORELIN ACETATE FOR PROSTATE CANCER]. Nihon Hinyokika Gakkai Zasshi. 2019;110(1):36-40. doi:10.5980/jpnjurol.110.36

51. Mark M, Thürlimann B. Fatal pneumonitis after treatment with pegylated liposomal doxorubicin in a patient with metastatic breast cancer in complete remission. Med Oncol. 2012;29(3):1477-1478. doi:10.1007/s12032-011-0002-1

52. Masago T, Watanabe T, Nemoto R, Motoda K. Interstitial pneumonitis induced by bicalutamide given for prostate cancer. Int J Clin Oncol. 2011;16(6):763-765. doi:10.1007/s10147-011-0239-x

53. Matsuoka H, Tsurutani J, Tanizaki J, Iwasa T, Komoike Y, Koyama A, et al. Regression of brain metastases from breast cancer with eribulin: a case report. BMC Res Notes. 2013;6:541. doi:10.1186/1756-0500-6-541

54. Mileshkin L, Prince HM, Rischin D, Zimet A. Severe interstitial pneumonitis following high-dose cyclophosphamide, thiotepa and docetaxel: two case reports and a review of the literature. Bone Marrow Transplant. 2001;27(5):559-563. doi:10.1038/sj.bmt.1702803

55. Morris MJ, Santamauro J, Shia J, Schwartz L, Vander Els N, Kelly K, et al. Fatal respiratory failure associated with treatment of prostate cancer using docetaxel and estramustine. Urology. 2002;60(6):1111. doi:10.1016/s0090-4295(02)01921-0

56. Murakami M, Kanemura H, Tomishima Y, Nakano E, Tamura T. Eribulin-induced Interstitial Pneumonia: A Case Series and Retrospective Cohort Study. Intern Med. 2020;59(4):563-567. doi:10.2169/internalmedicine.2779-19

57. Nagata S, Ueda N, Yoshida Y, Matsuda H, Maehara Y. Severe interstitial pneumonitis associated with the administration of taxanes. J Infect Chemother. 2010;16(5):340-344. doi:10.1007/s10156-010-0058-4

58. Nomura M, Sato H, Fujimoto N, Matsumoto T. Interstitial pneumonitis related to flutamide monotherapy for prostate cancer. Int J Urol. 2004;11(9):798-800. doi:10.1111/j.1442-2042.2004.00882.x

59. Okayasu K, Kawasaki T, Kumagai J, Miyazaki Y. Clinicoradiological course of abemaciclib-induced pneumonitis with histology findings. BMJ Case Rep. 2023;16(5). doi:10.1136/bcr-2022-254349

60. Okura F, Sato Y, Murakami E, Komatsu H, Yamamura Y, Ito Y. [A Case of Interstitial Pneumonitis Induced by Palbociclib]. Gan To Kagaku Ryoho. 2020;47(6):997-999.

61. Omoto H, Takada M, Fujii S, Ito H, Yamashita S. [A Case of Drug-Induced Interstitial Lung Disease Associated with Epirubicin and Cyclophosphamide Therapy before Operation]. Gan To Kagaku Ryoho. 2019;46(1):160-162.

62. Ostoros G, Pretz A, Fillinger J, Soltesz I, Dome B. Fatal pulmonary fibrosis induced by paclitaxel: a case report and review of the literature. Int J Gynecol Cancer. 2006;16 Suppl 1:391-393. doi:10.1111/j.1525-1438.2006.00222.x

63. Pankowska-Supryn M, Załęska M, Roszkowska-Śliż B, Roszkowski-Śliż K. Interstitial lung disease associated with docetaxel in a patient treated for breast cancer - a case report. Pneumonol Alergol Pol. 2015;83(5):378-382. doi:10.5603/PiAP.2015.0060

64. Pavlakis N, Bell DR, Millward MJ, Levi JA. Fatal pulmonary toxicity resulting from treatment with gemcitabine. Cancer. 1997;80(2):286-291. doi:10.1002/(sici)1097-0142(19970715)80:2%3C286::aid-cncr17%3E3.0.co;2-q

65. Pepels MJ, Boomars KA, van Kimmenade R, Hupperets PS. Life-threatening interstitial lung disease associated with trastuzumab: case report. Breast Cancer Res Treat. 2009;113(3):609-612. doi:10.1007/s10549-008-9966-8

66. Radzikowska E, Szczepulska E, Chabowski M, Bestry I. Organising pneumonia caused by transtuzumab (Herceptin) therapy for breast cancer. Eur Respir J. 2003;21(3):552-555. doi:10.1183/09031936.03.00035502

67. Rajendran A, Kunoor A, Pushpa Ragahavan R, Keechilat P. Ribociclib-associated organising pneumonia. BMJ Case Rep. 2021;14(12). doi:10.1136/bcr-2021-245187

68. Read WL, Mortimer JE, Picus J. Severe interstitial pneumonitis associated with docetaxel administration. Cancer. 2002;94(3):847-853. doi:10.1002/cncr.10263

69. Segura A, Yuste A, Cercos A, López-Tendero P, Gironés R, Pérez-Fidalgo JA, et al. Pulmonary fibrosis induced by cyclophosphamide. Ann Pharmacother. 2001;35(7-8):894-897. doi:10.1345/aph.10297

70. Shioi K, Yoshida M, Sakai N. Interstitial pneumonitis induced by bicalutamide and leuprorelin acetate for prostate cancer. Int J Urol. 2003;10(11):625-626. doi:10.1046/j.1442-2042.2003.00705.x

71. Shono M, Murakami K, Ohta M, Nakai H, Matsumura N. Interstitial lung disease caused by niraparib in ovarian cancer patient: a case report and literature review. Jpn J Clin Oncol. 2024;54(3):352-356. doi:10.1093/jjco/hyad171

72. Storaas E, Holmaas G, Gravdal K, Børretzen A, Eikesdal HP. Lethal pneumonitis after docetaxel chemotherapy: case report and review of the literature. Acta Oncol. 2013;52(5):1034-1038. doi:10.3109/0284186X.2012.750734

73. Sugiura-Nakane C, Taki Y, Suzuki R, Ikeda A, Igarashi T, Hasegawa S. [A Case of Interstitial Lung Disease That Developed after the First Administration of Trastuzumab-Deruxtecan in a Patient with Recurrent Breast Cancer]. Gan To Kagaku Ryoho. 2025;52(11):833-835.

74. Suratt BT, Lynch DA, Cool CD, Jones RB, Brown KK. Interferon-gamma for delayed pulmonary toxicity syndrome resistant to steroids. Bone Marrow Transplant. 2003;31(10):939-941. doi:10.1038/sj.bmt.1704032

75. Tamura A, Hashimoto M, Hosoi A, Hojo M. A case of eosinophilic bronchiolitis after the initiation of immune checkpoint inhibitor. Thorac Cancer. 2023;14(19):1894-1898. doi:10.1111/1759-7714.14931

76. Toyoshima M, Chida K, Suda T. [A case of early-onset cyclophosphamide-induced pneumonitis diagnosed by rechallenge test]. Nihon Kokyuki Gakkai Zasshi. 2009;47(12):1082-1086.

77. Tsukamoto N, Matsukuma K, Matsuyama T, Kashimura M, Kamura T, Uchino H, et al. Cyclophosphamide-induced interstitial pneumonitis in a patient with ovarian carcinoma. Gynecol Oncol. 1984;17(1):41-51. doi:10.1016/0090-8258(84)90058-1

78. Uğraklı M, Araz M, Demirkıran A, Çelik AF, Karakurt Eryılmaz M, Karaağaç M, et al. Pneumonitis associated with Trastuzumab emtansine in a patient with metastatic breast cancer. J Oncol Pharm Pract. 2022;28(3):740-745. doi:10.1177/10781552211066073

79. Vahid B, Mehrotra A. Trastuzumab (Herceptin)-associated lung injury. Respirology. 2006;11(5):655-658. doi:10.1111/j.1440-1843.2006.00907.x

80. Villalba-Cuesta PL, Álvaro-Vegue C, Carrasco-Muñoz CG, Gomis-Goti C, García-Villa A. Interstitial pneumonitis associated with leuprorelin acetate for a prostate cancer: A case report. J Oncol Pharm Pract. 2022;28(8):1910-1913. doi:10.1177/10781552221084058

81. Wieder JA, Soloway MS. Interstitial pneumonitis associated with neoadjuvant leuprolide and nilutamide for prostate cancer. J Urol. 1998;159(6):2099. doi:10.1016/S0022-5347(01)63270-1

82. Wong P, Leung AN, Berry GJ, Atkins KA, Montoya JG, Ruoss SJ, et al. Paclitaxel-induced hypersensitivity pneumonitis: radiographic and CT findings. AJR Am J Roentgenol. 2001;176(3):718-720. doi:10.2214/ajr.176.3.1760718

83. Yamamoto D, Yamamoto C, Yamamoto M. [A Case of Interstitial Pneumonitis Induced by Lapatinib plus Letrozole]. Gan To Kagaku Ryoho. 2016;43(12):2059-2061.

84. Yanagitani N, Shimizu Y, Kaira K, Tatsuno S, Sunaga N, Ishizuka T, et al. Pulmonary toxicity associated with vinorelbine-based chemotherapy in breast cancer. Gan To Kagaku Ryoho. 2008;35(9):1619-1621.

85. Yanai Y, Kosaka T, Hongo H, Oya M. Interstitial pneumonitis in a castration-resistant prostate cancer patient receiving cabazitaxel after thoracic radiation therapy: a case report. BMC Cancer. 2019;19(1):720. doi:10.1186/s12885-019-5942-4
